# Contribution of Intragenic DNA Methylation in Mouse Gametic DNA Methylomes to Establish Oocyte-Specific Heritable Marks

**DOI:** 10.1371/journal.pgen.1002440

**Published:** 2012-01-05

**Authors:** Hisato Kobayashi, Takayuki Sakurai, Misaki Imai, Nozomi Takahashi, Atsushi Fukuda, Obata Yayoi, Shun Sato, Kazuhiko Nakabayashi, Kenichiro Hata, Yusuke Sotomaru, Yutaka Suzuki, Tomohiro Kono

**Affiliations:** 1 Department of BioScience, Tokyo University of Agriculture, Tokyo, Japan; 2 Genome Research Center, NODAI Research Institute, Tokyo University of Agriculture, Tokyo, Japan; 3 Department of Maternal-Fetal Biology, National Research Institute for Child Health and Development, Tokyo, Japan; 4 Natural Science Center for Basic Research and Development, Hiroshima University, Hiroshima, Japan; 5 Department of Medical Genome Sciences, Graduate School of Frontier, The University of Tokyo, Kashiwa, Japan; The Babraham Institute, United Kingdom

## Abstract

Genome-wide dynamic changes in DNA methylation are indispensable for germline development and genomic imprinting in mammals. Here, we report single-base resolution DNA methylome and transcriptome maps of mouse germ cells, generated using whole-genome shotgun bisulfite sequencing and cDNA sequencing (mRNA-seq). Oocyte genomes showed a significant positive correlation between mRNA transcript levels and methylation of the transcribed region. Sperm genomes had nearly complete coverage of methylation, except in the CpG-rich regions, and showed a significant negative correlation between gene expression and promoter methylation. Thus, these methylome maps revealed that oocytes and sperms are widely different in the extent and distribution of DNA methylation. Furthermore, a comparison of oocyte and sperm methylomes identified more than 1,600 CpG islands differentially methylated in oocytes and sperm (germline differentially methylated regions, gDMRs), in addition to the known imprinting control regions (ICRs). About half of these differentially methylated DNA sequences appear to be at least partially resistant to the global DNA demethylation that occurs during preimplantation development. In the absence of *Dnmt3L*, neither methylation of most oocyte-methylated gDMRs nor intragenic methylation was observed. There was also genome-wide hypomethylation, and partial methylation at particular retrotransposons, while maintaining global gene expression, in oocytes. Along with the identification of the many *Dnmt3L*-dependent gDMRs at intragenic regions, the present results suggest that oocyte methylation can be divided into 2 types: *Dnmt3L*-dependent methylation, which is required for maternal methylation imprinting, and *Dnmt3L*-independent methylation, which might be essential for endogenous retroviral DNA silencing. The present data provide entirely new perspectives on the evaluation of epigenetic markers in germline cells.

## Author Summary

In mammals, germ-cell–specific methylation patterns and genomic imprints are established throughout large-scale de novo DNA methylation in oogenesis and spermatogenesis. These steps are required for normal germline differentiation and embryonic development; however, current DNA methylation analyses only provide us a partial picture of germ cell methylome. To the best of our knowledge, this is the first study to generate comprehensive maps of DNA methylomes and transcriptomes at single base resolution for mouse germ cells. These methylome maps revealed genome-wide opposing DNA methylation patterns and differential correlation between methylation and gene expression levels in oocyte and sperm genomes. In addition, our results indicate the presence of 2 types of methylation patterns in the oocytes: (i) methylation across the transcribed regions, which might be required for the establishment of maternal methylation imprints and normal embryogenesis, and (ii) retroviral methylation, which might be essential for silencing of retrotransposons and normal oogenesis. We believe that an extension of this work would lead to a better understanding of the epigenetic reprogramming in germline cells and of the role for gene regulations.

## Introduction

Throughout mammalian gametogenesis, dynamic DNA methylation changes occur in a sex- and sequence-specific manner. These changes result in the establishment of oocyte- and sperm-specific genomic imprints and unique methylation patterns of repetitive elements via DNA methyltransferase activity [1]–[4]. This process is indispensable for functional gamete and embryo development. For example, sex-specific methylation imprints are maintained throughout cell division after fertilization, despite genome-wide demethylation and *de novo* methylation during embryogenesis. These imprints control parent-of-origin specific monoallelic expression of a subset of genes, which are known as imprinted genes [5]–[9]. In addition, DNA methylation during spermatogenesis plays a crucial role in meiotic progression and retrotransposon silencing [10]–[14]. However, little is known about the profile and functional role of DNA methylation during oogenesis, except for the establishment of genomic imprints.

Recently, the epigenetic modifications which are responsible for regulating cell differentiation and embryo development have been studied in detail by using high-throughput sequencing: bisulfite sequencing (“BS-seq”); “Methyl-seq” with a methyl-sensitive restriction enzyme; “MeDIP-seq” with methylated DNA immunoprecipitation; and “MBD-seq” with a methyl-DNA binding domain protein antibody [15]–[26]. However, a major limitation of epigenomic studies is the lack of a standard methodology for DNA methylome analysis. Ideally, the gold standard is high resolution and genome-wide methylome analysis of germ cells. However, genome-wide methylome analysis of female germ cells has almost never been performed due to the limited availability of samples. Shotgun bisulfite sequencing (SBS) may be able to overcome this limitation and enable the determination of the cytosine methylation status of individual CpG sites at a whole-genome level without a bias toward CpG-rich regions [22], [23], [26] and with only relatively small-scale DNA samples [24], [27]. As a result, in this study, an improved SBS method for small-scale DNA samples was used to analyze the DNA methylome of mouse germ cells. In addition, the mouse germ cell transcriptome was investigated using high-throughput cDNA sequencing (mRNA-seq) to reveal relationships between DNA methylation and gene transcription in both male and female germ cells.

## Results

### Genome sequencing

We performed SBS analysis by using MethylC-seq [22] and a new SBS method called “whole bisulfitome-amplified DNA sequencing” (WBA-seq). The MethylC-seq and WBA-seq libraries were generated as shown in Figure S1. The MethylC-seq method generated 1010 and 1085 million tags (reads) from germinal vesicle (GV) stage oocytes and epididymal sperm, respectively. Oocyte DNA libraries generated by MethylC-seq showed higher redundancies than sperm DNA libraries. For example, 33.0% and 81.7% of the 21 million cytosines of CpGs in the mouse genome were covered by at least 1 sequence read from GV oocytes and sperm, respectively; whereas the average read depth (*i.e.*, the number of hits of reads that were mapped to a given position) was over 10× for both germ cells (Figure S2). The WBA-seq method generated 307 and 397 million tags from GV oocytes obtained from wild-type and *Dnmt3L*-deficient (*Dnmt3L^−/−^*) mice, respectively. WBA-seq libraries for GV oocytes showed higher genome coverage (60% of genomic CpGs were covered by at least 1 read) but with smaller average read depth (7.4×) than MethylC-seq library. Some reads from the oocyte libraries strongly matched mitochondrial DNA (mtDNA), satellite, low complexity, or simple repeat sequences (Figure S3), which might have been due to a distinct genomic copy number bias in the mitochondria of germ cells or an over-amplification bias. Thus, SBS results were simplified by removing the redundancy information (only mtDNA was separately examined for DNA methylation) and combining MethylC-seq and WBA-seq results for wild-type oocytes. Consequently, the average read depth was 18.8×, 4.4×, and 12.5× for wild-type and *Dnmt3L^−/−^* oocytes, and sperm, respectively, and 70.8%, 45.6%, and 79.9% of genomic CpGs were covered by at least 1 sequence read from each cell type (Table 1 and Figure S3). Furthermore, the average read depths of MethylC-seq of mouse blastocysts and embryonic stem cells (ESCs), which served as zygote and stem cell controls, were 12.8× and 6.1×, respectively (Table 1).

**Table 1:** Summary of shotgun bisulfite sequencing data.

Sample	Method	Aligned tags (base)	Genome covarage		Read depth
			(>x1)	(>x5)	
Wild-type oocyte	MethylC-seq & WBA-seq	51,166,451,066	70.8%	39.4%	18.8
*Dnmt3L^−/−^* oocyte	WBA-seq	11,872,662,647	45.6%	19.6%	4.4
Sperm	MethylC-seq	34,153,237,944	79.9%	63.4%	12.5
Blastocyst	MethylC-seq	34,857,014,339	86.2%	79.4%	12.8
ESC	MethylC-seq	16,691,289,063	73.0%	38.9%	6.1

### Methylome of mouse germ cells

**Figure 1:**
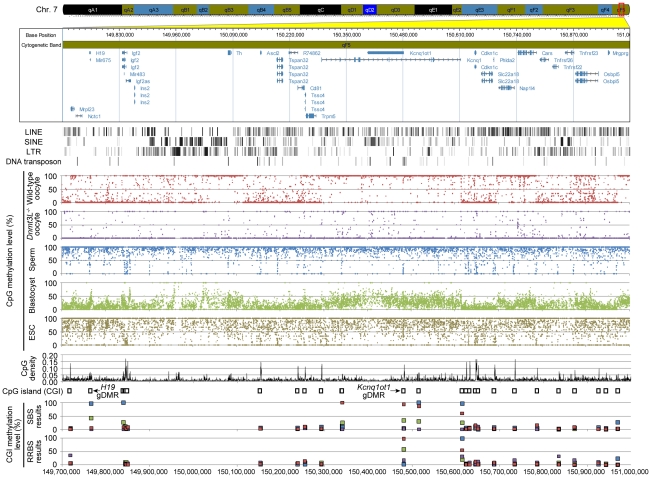
High-resolution DNA methylome map of mouse distal chromosome 7 imprinting cluster. Illumina GenomeStudio viewer displays the locations of genes in distal chromosome 7 (149,700,000–151,000,000). Black vertical bars represent the location of 4 repetitive elements: LINE, SINE, LTR, and DNA transposons. Red, purple, blue, green, and khaki dots represent the methylation levels at individual CpGs in wild-type oocyte, *Dnmt3L* ^−/−^ oocyte, sperm, blastocyst, and ESC genomes, respectively. Black line plots depict the distribution of CpG densities (number of CpG per 200 nt) of individual CpGs. Open boxes represent the location of CpG islands (CGIs). Red, purple, blue, and green boxes represent the methylation levels at individual CGIs in wild-type oocyte, *Dnmt3L* ^−/−^ oocyte, sperm, and blastocyst genomes, respectively, determined by our results from shotgun bisulfite sequencing (SBS) method and Smallwood's results from reduced representation bisulfite sequencing (RRBS) method [38].

**Figure 2:**
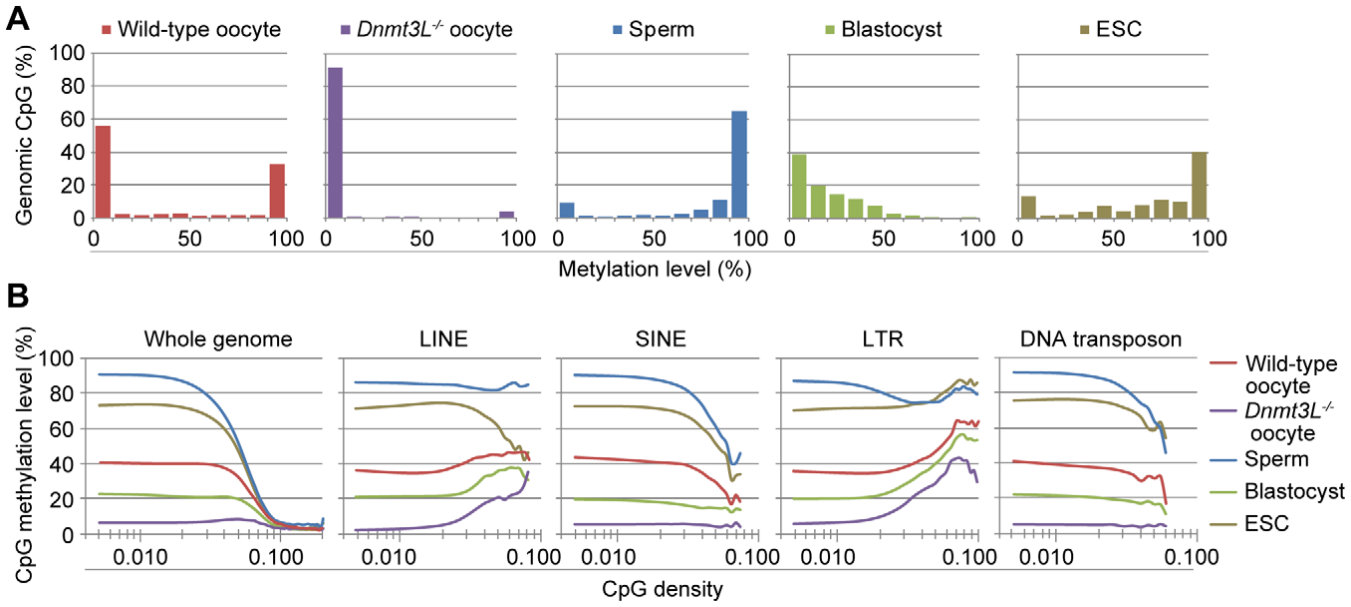
Genome-wide methylation profiling of mouse germ cells. (A) Histograms of methylation levels of genomic CpGs in wild-type oocyte, *Dnmt3L^−/−^* oocyte, sperm, blastocyst, and embryonic stem cell (ESC) genomes. (B) CpG methylation levels are plotted as a function of CpG density for the whole genome and 4 families of transposable elements (long interspersed nuclear element (LINE), short interspersed nuclear element (SINE), long terminal repeat (LTR), and DNA transposon).

The average methylation level of wild-type oocytes (40.0%) was less than half that of sperm (89.4%) (Figure S4). This difference in global DNA methylation between male and female germ cells was consistent with results from the previous studies [28], [29]. The *Dnmt3L^−/−^* oocyte genome was observed to be hypomethylated, exhibiting a methylation level of only 5.5%. Furthermore, blastocysts showed a lesser extent of methylation (21.3%) than did wild-type oocytes; ESCs, on the other hand, showed relatively high levels of methylation (70.6%). To elucidate the distribution of methylation levels on CpG sites, on regional and genome-wide scales, we created dot plots of CpG methylation for individual chromosomes and histograms of the methylation levels for all CpGs. These graphs revealed that hypermethylated CpGs in oocytes tended to cluster in transcribed regions of particular genes (*e.g.*, *Kcnq1* or *Rlim* genes, known to be expressed in oocytes [30], [31]); the sperm genome was almost entirely hypermethylated, except at most CpG-rich regions (Figure 1 and Figure S5). Specifically, 55.7% of the CpGs in the oocyte genome exhibited <10% methylation, whereas another 32.0% of CpGs exhibited ≥90% methylation (Figure 2A). The *Dnmt3L^−/−^* oocyte genome was also hypomethylated in almost all chromosomal regions (Figure S6). The methylation level of the mtDNA genome in *Dnmt3L^−/−^* oocytes (4.4%) was lower than that observed in wild-type oocytes (6.6%). Sperm methylation levels, by comparison, were relatively high (14.7%), whereas those of the blastocysts and ESCs were quite low (1.3% and 2.1%, respectively) (Figure S4).

Since previous studies revealed a significant correlation between CpG frequency and methylation within intra- and intergenic regions in somatic cells [32], [33], the CpG density and methylation levels were compared to identify genome-wide differential methylation patterns in germ cells. CpG density was defined as the number of CpG dinucleotides in 200 nucleotide (nt) windows (*e.g.*, 1 CpG dinucleotide per 200 nt corresponds to a density of 0.005). At low CpG densities (range, 0.005–0.05), the oocyte genome was about 50% methylated, whereas the sperm genome was 80–90% methylated. At moderate to high CpG densities (range, 0.05–0.2), both male and female germ cells were hypomethylated (Figure 2B). Furthermore, 4 families of transposable elements (long interspersed nuclear elements (LINEs), short interspersed nuclear elements (SINEs), long terminal repeats (LTRs), and DNA transposons) were moderately methylated in oocyte genomes but were hypermethylated in sperm. In addition, a general trend towards higher methylation levels at higher CpG densities in the oocyte genome occurred in LTRs. Conversely, a trend toward lower CpG methylation levels at higher CpG densities in the wild-type oocyte and sperm genomes was observed in SINEs and DNA transposons. In contrast, all of these transposable elements were hypomethylated in *Dnmt3L^−/−^* oocytes. Interestingly, however, there was partial CpG methylation in LINEs and LTRs at relatively high CpG densities (range, 0.03–0.1). These complete or partial undermethylations were confirmed by bisulfite sequencing in L1 LINEs, B1/Alu SINEs, and intracisternal A particle (IAP) LTRs (Figure S7). These results suggested that each germ cell has a unique sequence- and CpG-density-dependent methylation pattern. In addition, oocyte CpG methylation, except in a subset of retrotransposons, appears to be *Dnmt3L* dependent.

**Figure 3:**
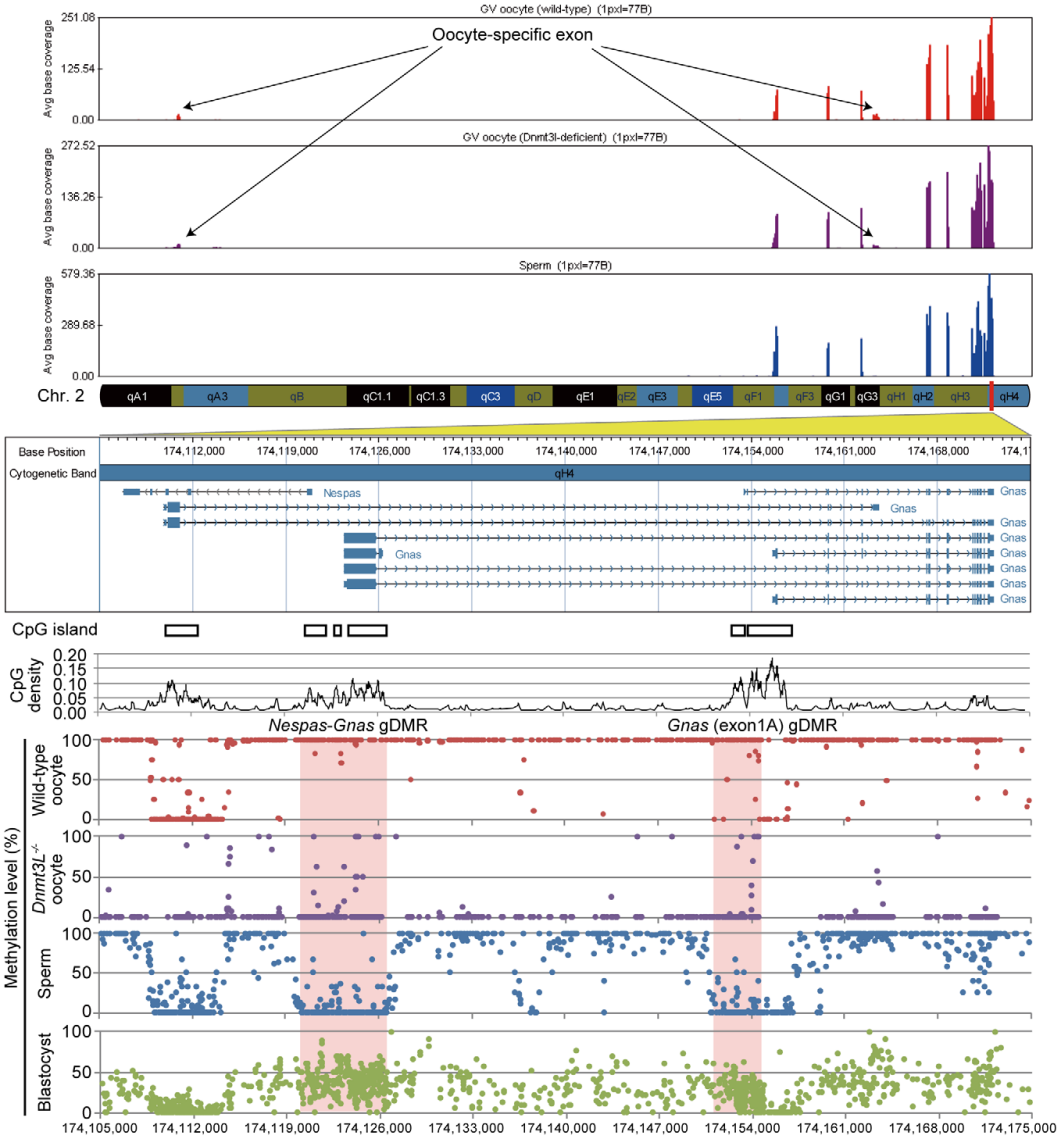
High-resolution genome-wide mRNA expression and CpG methylation profiling. GenomeStudio view of mRNA-seq data and CpG methylation map of the genomic region spanning the *Nespas*-*Gnas* maternally imprinted locus. (*Top*) Genomic stacked alignment plots of wild-type oocytes, *Dnmt3L^−/−^* oocytes, and sperm. (*Middle*) Open boxes and black line plots represent the location of CGIs and the distribution of CpG densities of individual CpGs, respectively. (*Bottom*) Red, purple, blue, and green dots represent the methylation levels at individual CpGs in wild-type oocyte, *Dnmt3L* ^−/−^ oocyte, sperm, and blastocyst genomes, respectively. The red shaded areas show the extent of two maternal imprinting control regions (ICRs).

We also characterized the methylation patterns of 15 germline-differentially methylated regions (gDMRs). The differential (between oocyte and sperm) methylation occurs at imprinted gene loci (also called imprinting control regions (ICRs)). The ICRs of maternally methylated imprinted genes (*e.g.*, *Nespas-Gnas*) were shown to be hypermethylated in oocytes but hypomethylated in sperm, while the converse was true in ICRs of paternally-methylated imprinted genes (*e.g.*, *H19*) (Figure 3 and Figure S8). Interestingly, only the *Snrpn* gDMR was partially methylated (35.7%), whereas all other maternal ICRs were hypomethylated in *Dnmt3L^−/−^* oocytes (Table 2). This residual methylation might result in the stochastic acquisition of the maternal imprint in the progeny of *Dnmt3L^−/−^* females [34]. These results strongly suggested that the methylation level of individual CpGs can be determined from DNA methylome maps with a high degree of accuracy.

**Table 2:** CpG methylation profiling of 12 maternal and 3 paternal imprinting control regions.

	Gene locus	Chr.	Extents of the ICRs†		Average methylation levels				
			Start	End	Wild-type oocyte	*DnmtLl^−/−^* oocyte	Sperm	Blastocyst	ESC
*Maternally methylate imprinted genes*	*Nespas-Gnas*	2	174,119,863	174,126,564	99.3%	5.6%	3.9%	38.2%	55.9%
	*Gnas* (exon1A)	2	174,150,877	174,154,638	95.2%	3.5%	4.1%	20.4%	7.8%
	*Peg10*	6	4,696,743	4,699,483	95.9%	6.7%	5.5%	31.8%	57.1%
	*Mest*	6	30,684,932	30,689,966	96.5%	2.3%	4.2%	30.7%	52.6%
	*Peg3*	7	6,679,787	6,684,257	98.1%	3.0%	2.5%	32.1%	42.8%
	*Snrpn*	7	67,147,381	67,151,583	94.1%	35.7%	4.6%	34.3%	64.9%
	*Kcnq1ot1*	7	150,480,736	150,482,810	97.9%	2.2%	4.3%	34.1%	52.0%
	*Plagl1*	10	12,809,697	12,812,131	99.9%	1.3%	7.4%	35.4%	53.0%
	*Grb10*	11	11,925,127	11,927,100	98.0%	1.2%	5.3%	38.5%	78.7%
	*Zrsr1*	11	22,871,610	22,874,212	94.1%	5.2%	6.8%	34.8%	47.0%
	*Igf2r*	17	12,934,169	12,935,816	99.1%	0.9%	3.8%	44.2%	53.2%
	*Impact*	18	13,130,435	13,133,510	97.2%	2.4%	6.6%	43.1%	38.6%
*Paternally methylated imprinted genes*	*H19*	7	149,764,673	149,771,930	13.5%	0.6%	96.5%	40.8%	65.5%
	*Rasgrf1*	9	89,767,090	89,775,128	7.4%	0.7%	92.0%	25.2%	59.4%
	*Dlk1-Meg3*	12	110,762,703	110,773,093	18.9%	0.9%	96.8%	32.4%	83.1%

**†:** : The extents of each region in germ cells were determined by bisulfite sequencing study [39].

The study of mammalian DNA methylation patterns has previously suggested that methylation predominantly occurs at CpG sites; however, more recent studies, based on SBS methods, have indicated that methylation at non-CpG sites also occurs in human ESCs [22], [23]. Detection of non-CpG methylation is one of the applications of the bisulfite-based methylation analysis but is problematic due to the incomplete conversion of cytosine, and overestimates of such cytosine by PCR amplification, which cannot be discriminated from true methylation. In order to evaluate the methylation status of non-CpG sites and avoid these problems, additional SBS analysis of mouse GV oocytes, sperm, blastocysts, and ESCs was performed by a non-amplification technique, termed Post-Bisulfite Adapter Tagging (PBAT) [Miura F. & Ito T, personal communication]. All C (originally methylated cytosine) and T (originally unmethylated cytosine) that mapped to genomic CpG and CpH sites (H = A, T, or C) were counted. The PBAT results showed CpG methylation ratios (C ratios = 0.395, 0.748, 0.137, 0.615 in oocytes, sperm. blastocysts, and ESCs) which are similar to the average methylation levels of individual DNA methylome maps obtained by MethylC-seq and WBA-seq among all examined cells. Interestingly, a relatively high fold enrichment of non-CpG methylation was observed in GV oocytes (C ratio = 0.034–0.038), but not in the other cell types, including mouse ESCs (C ratio <0.01) (Figure S11).

### Relationship between the DNA methylome and transcriptome of mouse germ cells

**Figure 4:**
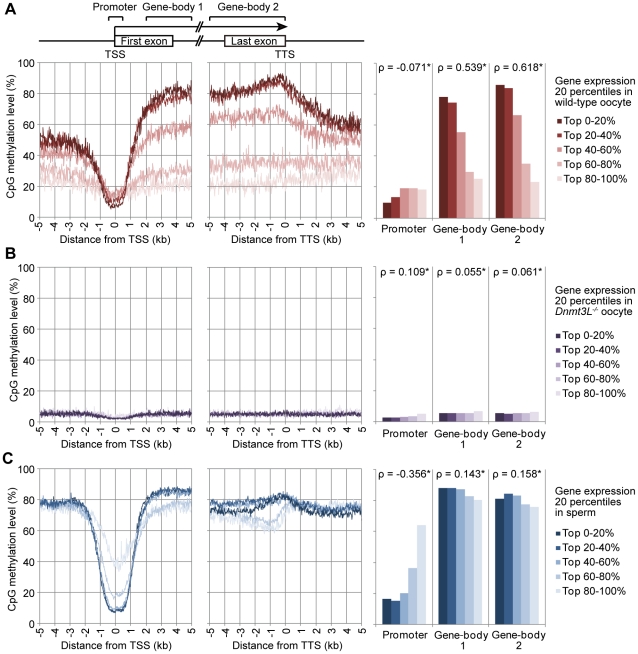
Relationship between gene expression and methylation in promoter and gene-body regions in mouse germ cells. The expression level of genes in wild-type oocytes (A), sperm (B), and *Dnmt3L^−/−^* oocytes (C) were divided into 5 percentile groups. The distribution of methylation is shown ±5 kb from the transcription termination site (TTS; *left*) and transcription start site (TSS; *middle*). The graphs on the right show the average methylation levels in the promoter and gene-body regions. Spearman's rank correlation coefficient (ρ) was used to test the statistical significance of the correlation between gene expression and DNA methylation levels (*: p<1×10^−9^).

To elucidate the interaction between intragenic DNA methylation and gene transcription, the correlation between promoter and gene-body methylation and expression levels for 20,854 different genes was examined. The mRNA-seq profiles for germ cells and ESCs are shown in Table S1. The results showed that mRNA transcript levels in oocytes were strongly correlated to gene-body methylation levels (Spearman's ρ>0.5, *p*<1×10^−9^) but were not significantly correlated to promoter methylation levels (|ρ|<0.1) (Figure 4A). For example, the regions +2 to +5 kb from the transcription start site (TSS) and 0 to −5 kb from the transcription termination site (TTS) were hypermethylated (60–90% methylation) for the top 20% of expressed genes but were hypomethylated (10–30% methylation) for the bottom 20% of expressed genes. However, areas near the TSS (±500 base pairs (bp)) were hypomethylated (10–20% methylation) in all genes, regardless of their expression level. In contrast, in the *Dnmt3L^−/−^* oocyte genome, the correlation between gene expression and gene-body methylation was very weak (|ρ|<0.1) (Figure 4B). In the sperm genome, promoter methylation was negatively correlated (Spearman's ρ = −0.36, p<1×10^−9^) with gene expression, whereas gene-body methylation was positively correlated (Spearman's ρ = 0.14–0.16, p<1×10^−9^) to gene expression; the latter correlation was weaker than that observed in the oocyte genome (Figure 4C).

### Role of *Dnmt3L* in the DNA methylome/transcriptome relationship

**Figure 5:**
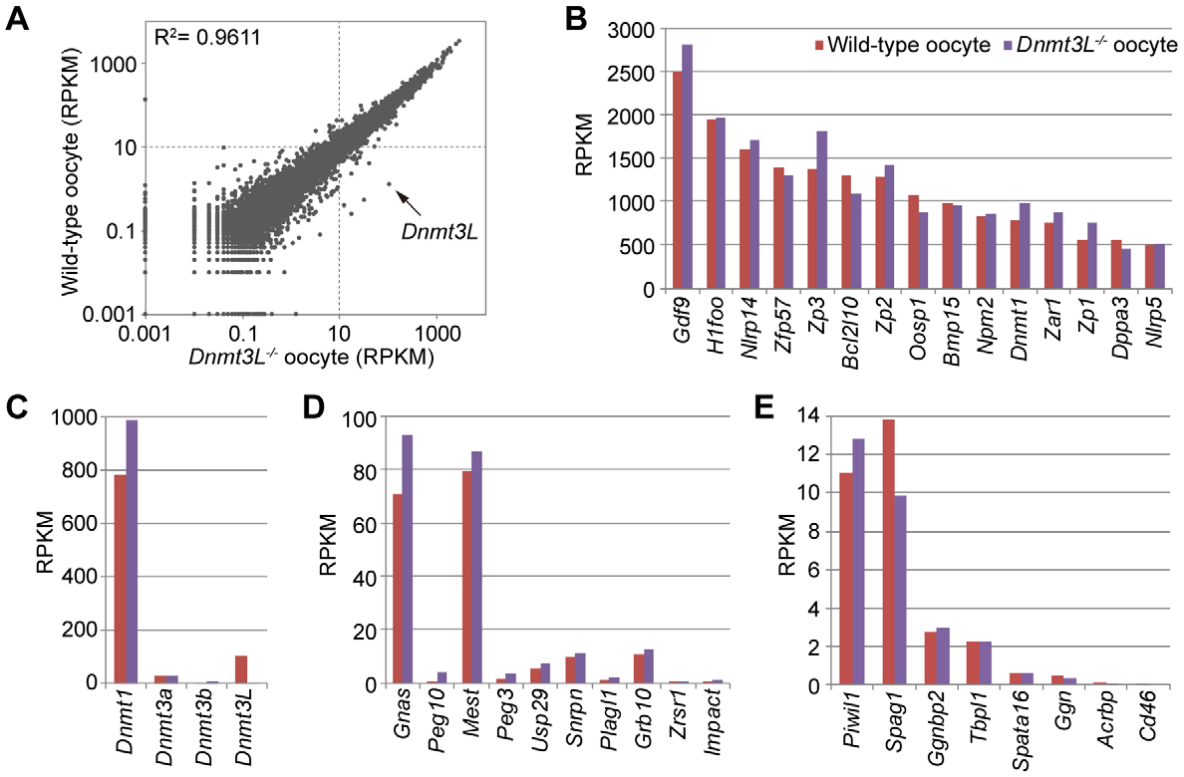
Comparison of gene expression profiles between wild-type and *Dnmt3L^−/−^* oocytes. (A) Scatter plot and correlation coefficient (R^2^) of RPKM values of 20,854 genes in wild-type and *Dnmt3L^−/−^* oocytes. Expression levels of oocyte-specific genes (B), DNA methyltransferase genes (C), maternally-imprinted genes that are potentially necessary to establish methylation imprints (D), and male germline-specific genes that contain oocyte-specific methylated CpG islands (CGIs) (E).

Further investigation of gene expression patterns in oocyte genomes revealed that the mRNA transcript levels between wild-type and *Dnmt3L^−/−^* oocytes were very highly correlated (R^2^ = 0.9611) (Figure 5A). In fact, there were no significant differences in the expression levels of representative oocyte-specific genes (*e.g.*, *Gdf9*, *Bmp15*, *Bcl2l10*, *Zp1*, *Zp2*, *Zp3*, *Zar1*, *Npm2*, *Nlrp5*, and *Dppa3*, which are responsible for ovarian follicle formation, reproduction, and early development [35]) and DNA methyltransferase genes (*e.g.*, *Dnmt1*, a maintenance methyltransferase, and *Dnmt3a* and *Dnmt3b de novo* methyltransferases); the expected difference in the expression level of *Dnmt3L* between wild-type and *Dnmt3L^−/−^* oocytes was observed (Figure 5B, 5C). These results suggested that changes in gene expression did not occur during oogenesis, despite global intragenic hypomethylation in *Dnmt3L^−/−^* oocytes. Furthermore, the expression levels and exon patterns of maternally-methylated imprinted genes across each ICR were not altered in *Dnmt3L^−/−^* oocytes (Figure 3 and Figure 5D). This result suggested that the disruption of maternal methylation imprints in the *Dnmt3L^−/−^* oocyte genome was not due to the lack of their transcription [36]. On the other hand, maternal methylation imprints at ICRs (and many other hypermethylations at transcribed regions) in wild-type oocyte genomes might be the result of gene transcription via *Dnmt3L*-mediated intragenic methylation.

Surprisingly, gene expression in ESC genomes was negatively correlated with promoter methylation and was not positively correlated with gene-body methylation (Figure S12). Meanwhile, these ESCs showed the apparent expression of all DNA methyltransferase gene families including *Dnmt3L* (Figure S13). Previous studies indicated that the zygotic and somatic functioning of *Dnmt3L* is not essential for global methylation in ESCs in mice [6]. Thus, unlike oocytes, the functional role of *Dnmt3L* in gene-body methylation after fertilization is unclear. However, the expression of pluripotency-associated genes, *Pou5f1*, *Klf4*, *Sox2*, *Myc*, *Nanog*, and *Lin28a*, was clearly observed in ESCs. The expression of *Pou5f1*, *Lin28a*, and *Glis1*, recently identified as maternal reprogramming factors, were also observed in oocytes (Figure S14). While differential expression of the pluripotency genes among germ and stem cells was observed, the promoter regions of these genes demonstrated low-level methylation in almost all of the examined cells. In sperm cells, only the *Nanog* promoter was hypermethylated (this result was similar to a previous study [29]).

### Identification and characterization of germline differentially methylated regions

**Figure 6:**
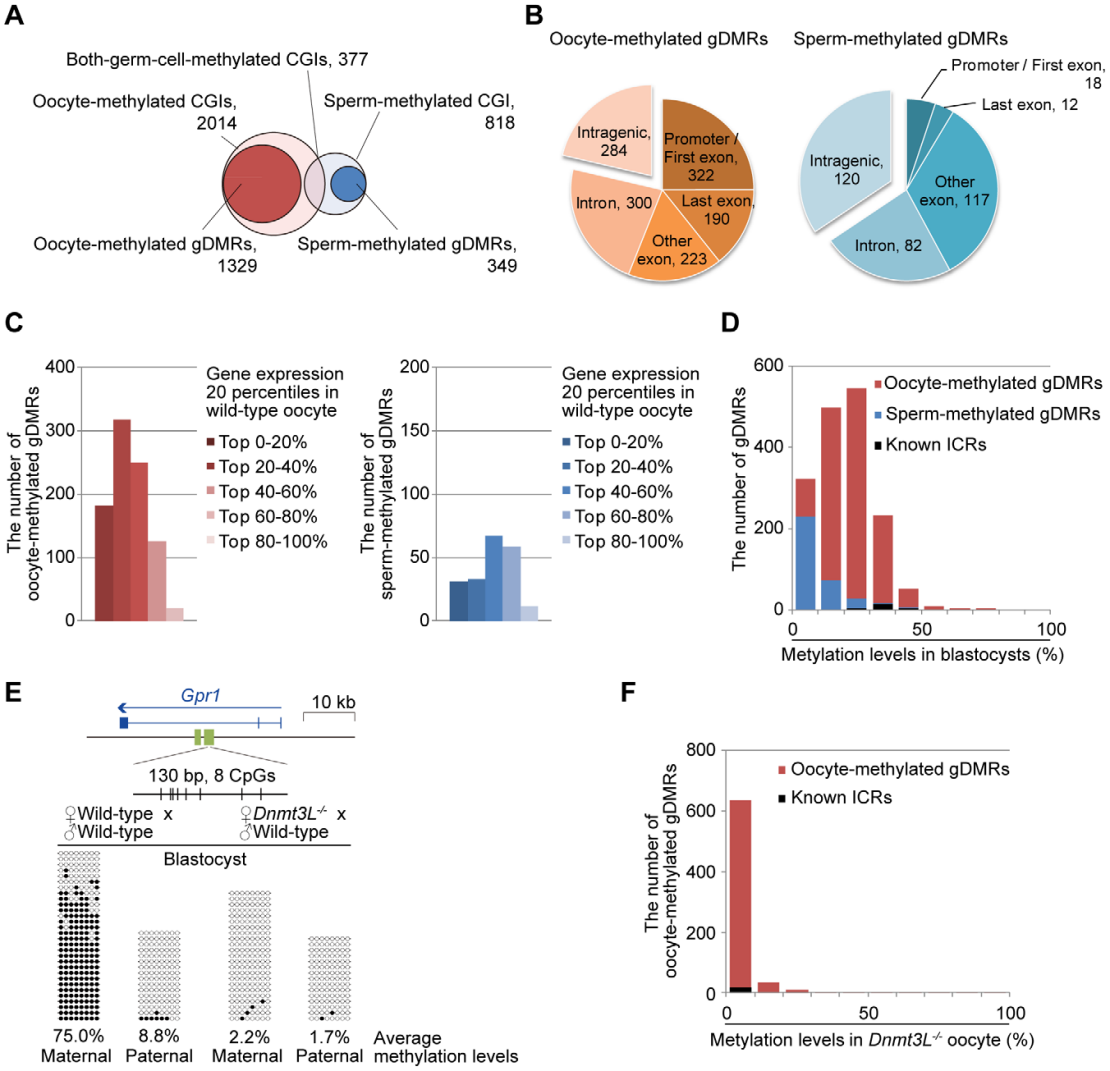
Identification of germline differentially methylated CGIs from DNA methylome profiles. (A) Venn-like diagram of two groups of CGIs, namely, oocyte-methylated CGIs (light pink) and sperm-methylated CGIs (light blue) and two groups of gDMRs, namely, oocyte-methylated gDMRs (red) and sperm-methylated gDMRs (blue). (B) The genomic distribution of 1329 oocyte-methylated (left) and 349 sperm-methylated gDMRs (right). The gDMRs were classified into 5 genomic locations; promoter (within 500-bp upstream from the first exon) or first exon, last exon, other exon, intron, and intergenic region. (C) The locations of the intragenic 1045 oocyte-methylated (left) and 229 sperm-methylated gDMRs (right). The gDMRs were classified into 5 gene group locations; the genes were divided into 5 percentile groups according to their expression levels in wild-type oocytes and sperm, as shown in Figure 3. (D) Histograms of the methylation levels of the gDMRs in blastocysts. The number of newly identified oocyte-specific, sperm-specific methylated gDMRs, and known ICRs are shown in black, red, and blue, respectively. (E) Bisulfite sequencing at the *Gpr1* gDMR in mouse blastocysts. (*Top*) Schematic representation of paternally-expressed *Gpr1*. The gene and gDMRs are shown in blue and green, respectively, and CpG sites are represented by vertical bars. (*Bottom*) Methylated and unmethylated CpGs are indicated by open and closed circles, respectively. The maternal and paternal alleles were distinguished by three polymorphisms between C57BL/6N and JF1 mice (G/A at 63,247,064; T/A at 63,247,072; and TA/AG at 63,247,089–63,247,090 on chromosome 1). (F) Histograms of the methylation levels of the demethylation-resistant oocyte-methylated gDMRs in *Dnmt3L^−/−^* oocytes. The number of newly identified oocyte-specific, sperm-specific methylated gDMRs, and known ICRs are shown in black, red, and blue, respectively.

To identify gDMRs, the average CpG methylation levels of individual CpG islands (CGIs), which are CpG-rich genomic regions often lacking DNA methylation, were calculated. Recently, Illingworth et al. determined the number of CGIs by deep sequencing of isolated, unmethylated DNA clusters [37]. Among the 23,021 mouse CGIs (22,974 CGIs were informative in both oocytes and sperm), 2014 were highly methylated (≥80% methylation) in oocytes, 818 were highly methylated in sperm, and 377 were highly methylated in both germ cells (Figure 6A). Furthermore, we also identified 1678 gDMRs (≥80% methylation in 1 gamete and ≤20% in the other), 1329 of which were oocyte-specific methylated CGIs, while the remaining 349 were sperm-specific methylated CGIs (Figure 6A, Figure S6, and Table S2). Among these gDMRs, 646 gDMRs were confirmed to show a differential methylation status between GV oocytes and sperm (by similar criteria: ≥75% methylation in 1 gamete and ≤25% in the other); the methylation status was previously examined by performing large-scale bisulfite sequencing of CpG-rich regions of the genome (reduced representation bisulfite sequencing: RRBS) (Table S3) [38]. Additionally, almost all known ICRs except *Zdbf2* DMRs (which do not have any CGIs) were re-identified from our gDMR list (Table S2).

A total of 78% oocyte-methylated gDMRs (n = 1045) were located within the intragenic regions. Approximately 25% of the oocyte-methylated gDMRs (n = 322) overlap with either the first exon or the proximal promoter regions of the genes, as has been observed with most of the described maternal ICRs [39]; only 5% of the sperm-methylated gDMR (n = 18) showed such overlap. Alternatively, 34% of sperm-methylated gDMRs (n = 120) overlap with intergenic regions, as in all known paternal ICRs (Figure 6B). Interestingly, oocyte-methylated gDMRs in transcribed regions tended to be more abundant within highly expressed genes, but such a trend was not observed in the sperm genome (Figure 6C). Oocyte-methylated gDMRs were also identified in non-imprinted genes, such as the DNA methyltransferase genes (*e.g.*, *Dnmt1* and *Dnmt3b*) and some male germline-specific genes (*e.g.*, *Piwil1*, *Spag1*, *Ggnbp2*, *Tbpl1*, *Spata16*, *Ggn*, *Acrbp*, and *Cd46*). The oocyte-methylated gDMR in *Dnmt1* was located in spermatocyte- and somatic-specific exons, while oocyte-specific exons were hypomethylated in oocytes (Figure S9). *Dnmt3L^−/−^* oocytes also showed hypomethylation in most of these gDMRs. Significant changes in the expression levels of genes with alternative splicing patterns were not observed in the *Dnmt3L^−/−^* oocyte genome (Figure 3, Figure 5E, and Figure S9). These results indicate that these oocyte-specific methylated gDMRs do not regulate gene expression or alternative splicing during the oocyte stage.

To determine whether or not these germ cell-specific methylations are maintained after fertilization, when the genomes undergo global demethylation, the individual CGI methylation levels in blastocyst genomes were calculated. In blastocysts, all ICRs demonstrated low to moderate methylation (25.1–64.3%), whereas many gDMRs were demethylated (0–20%) (Figure 6D). Furthermore, 817 oocyte-methylated gDMRs (including *Piwil1*, despite being a non-imprinted gene locus) and 34 sperm-specific gDMRs were resistant to demethylation during early embryogenesis (≥20% methylation in blastocysts) (Figure 6D and Table S2). Among the demethylation-resistant gDMRs, a novel gDMR in the intron of *Gpr1* (Figure S10) was found to be a tissue-specific, paternally-expressed imprinted gene [40]. Bisulfite sequencing analysis showed that this gDMR was hypomethylated in *Dnmt3L^−/−^* oocytes and maternal allele-specific methylation was detected in this region in blastocysts (Figure 6E). Methylation profiles in ESCs showed that 26% (n = 213) of the demethylation-resistant gDMRs became less methylated (0–20%) whereas the other gDMRs maintained or increased DNA methylation (Figure S15). Among ICRs, only *Gnas* exon1A ICR was demethylated (7.8%), whereas the other ICRs developed partial or high methylation levels (range, 38.6–83.1%) in ESCs (Table 2). Among other demethylation-sensitive gDMRs, which were demethylated (<20% methylation) in blastocysts, many (76%, n = 264) sperm-methylated gDMRs were re-methylated (≥20% methylation); most (81%, n = 416) of the oocyte-methylated gDMRs maintained low methylation (0–20%) in ESCs (Figure S15). Finally, out of 704 demethylation-resistant (in blastocysts) oocyte-methylated gDMRs which were informative in *Dnmt3L^−/−^* oocytes, only 4 remained hypermethylated (80–100% methylation) in the *Dnmt3L^−/−^* oocyte genome. However, almost all other oocyte-specific methylation marks at gDMRs were *Dnmt3L*-dependent (Figure 6F). These results suggest that *Dnmt3L*-mediated methylation during oogenesis regulates the establishment of most heritable oocyte-specific marks, including genomic imprints.

## Discussion

To the best of our knowledge, this is the first study to generate single-base resolution maps of DNA methylomes spanning the entire genome of mouse germ cells. The oocyte maps are particularly valuable and informative because, in the past, such an analysis was prohibitive due to the need for large quantities of DNA. Recently, Smallwood et al. [38] reported large-scale DNA methylation patterns in mouse germ cells by using the RRBS method, which targets only CpG-rich regions. However, our more comprehensive results provide strong evidence that gene expression was positively correlated to *Dnmt3L*-dependent intragenic methylation in oocytes, and that methylation patterns in oocytes differed from those in sperm and non-germline cells.

The functional role of gene-body methylation has been an enigma despite its conservation in plants and animals [41]–[43]. Maunakea et al. [44] suggested that gene-body methylation is involved in the regulation of alternative splicing events. Although methylated gDMRs were detected in the alternative exons of *Dnmt1* and *Gnas* in mouse oocytes, loss of oocyte-specific methylation marks in the *Dnmt3L^−/−^* oocytes did not affect the expression patterns of alternatively spliced transcripts. Therefore, our results indicate that gene-body methylation is not involved in alternative splicing in oocytes.

Previously, Chotalia et al. [36] showed that transcription during the oocyte stage is required for the establishment of maternal methylation marks on an imprinted gene. The present results show that *Dnmt3L^−/−^* oocytes lost almost all of their maternal methylation imprints while maintaining a constant amount of mRNA through each ICR despite the global loss of intragenic methylation. Thus, these results strongly suggest that the establishment of genomic imprints via transcription is mediated by *Dnmt3L*-dependent intragenic methylation.

A possible mechanism for gene-body methylation involves the exposure of intragenic regions to DNA methyltransferases, considering that RNA polymerase disrupts the chromatin structure during transcription. However, not all transcripts across gDMRs corresponded to highly expressed genes in oocytes (Figure 6C). Therefore, other epigenetic marks with an open chromatin structure might also be important for DNA methylation in oocytes. For instance, a recent knockout study showed that *Kdm1b*, which encodes histone H3K4 demethylase, is required for the establishment of some maternal methylation imprints [45]. Thus, several factors, including transcriptional and epigenetic modifications, might be involved in *Dnmt3L*-mediated intragenic methylation.

The results of this study show that gene-body methylation was correlated to gene expression in sperm. However, the extent of that correlation is much less than in oocytes due to genome-wide hypermethylation, including in low-CpG-density regions. In male germline cells, global methylation acquisition begins during late embryonic development and before birth [3]. To more clearly show this correlation, analysis of early-stage germ cells in fetal or neonatal animals might be required. Surprisingly, a positive correlation between mRNA expression and gene-body methylation was not observed in mouse ESCs. In addition, the accumulation of non-CpG methylation was not observed in mouse ESCs. These results contradict the results of another study, which showed that active transcription was associated with intragenic DNA methylation with non-CpG methylation in human ESCs [22], [23]. This discrepancy might reflect the differences between human and mouse ESCs, the precise cell derivations or culture conditions [46], [47]. However, further comparative studies on germ cell epigenomes from other species are required to further elucidate the functional role of epigenetic marking systems.

In this study, a large number of heritable oocyte-specific methylation marks were identified within a set of novel CpG islands [37]. The difference in the number of oocyte- and sperm-specific gDMRs reflects the fact that only 3 or 4 paternally-methylated imprinted loci were observed, as compared to approximately 20 maternally-methylated imprinted loci. The reason for the relative abundance of oocyte-specific methylated CGIs might be related to the intragenic methylation of CpG-rich regions, which are hypomethylated in sperm. The results show that most of the oocyte-specific marks are *Dnmt3L*-dependent, similar to results recently obtained by RRBS-based analysis [38]. However, whether all of these CpG-rich regions serve as imprinting methylation marks is unclear. For instance, although many genes with oocyte-specific methylation marks were identified (Figure 6B), the evidence that these genes were imprinted was lacking (*e.g.*, *Piwil1* and *Dnmt1*). These methylation marks might not be involved in the formation of a fertile oocyte but might play crucial roles in gene expression after fertilization. Furthermore, ESC methylomes showed that many gDMRs, especially sperm-specific gDMRs, acquired new methylation patterns after implantation. Methylation of these CGIs might control tissue-specific gene expression [48], [49]. Partial alternation of imprinted methylation patterns in ESCs were observed in the present study, potentially caused by significant differences in the extent of the ICRs during embryo development [39]. A fuller understanding of epigenetic stability will require further methylome profiling during early embryogenesis and stem cell differentiation. The present study also identified a gDMR as a novel ICR candidate in the intron of the imprinted *Gpr1* gene. Thus, traditional promoter arrays may not identify all ICRs. However, further analyses are needed to determine which gDMRs, identified in the CpG methylome maps, are true ICRs at the imprinted *Gpr1*-*Zdbf2* locus [40], [50].

mRNA-seq results showed that the expression levels of most genes in the wild-type and *Dnmt3L^−/−^* oocytes were similar. For instance, the expression level of almost all oocyte-specific genes, which regulate ovarian follicle formation, reproduction, and early development, were not significantly altered (Figure 5B and Table S1). These results are consistent with the findings of previous studies, which showed that *Dnmt3L^−/−^* female mice were capable of producing fertile oocytes (however, their offspring were not viable due to the lack of imprinting) [5], [6]. Thus, regulation of oocyte-specific genes must be beyond the control of *Dnmt3L*-dependent cytosine methylation.

Although *Dnmt3L^−/−^* oocytes showed global hypomethylation at low to high CpG densities, some families of retrotransposons, such as LINEs and LTRs, were partially methylated at moderate to high CpG densities. Therefore, *Dnmt3L*-independent methylation might be involved in the silencing of retrotransposons and completion of oocyte meiosis. Previously, De La Fuente et al. [51] showed that *Hells* (also known as *Lsh*), which encodes a member of the sucrose non-fermenter 2 (SNF2) family of chromatin remodeling proteins, is required for DNA methylation of IAP and pericentromeric satellite repeats as well as repression of IAP retrotransposition in pachytene oocytes. Unfortunately, measurement of the methylation levels of satellite DNA, which is abundant in the pericentromeric regions, was not possible because these sequences were excluded from our analysis. However, a previous sequencing study showed that methylation levels of satellite DNA did not differ between the wild-type and *Dnmt3L^−/−^* oocytes [52]. Combined, these results suggest the presence of 2 types of oocyte methylation patterns: (i) *Dnmt3L*-mediated intragenic methylation that is essential for early embryogenesis and (ii) *Dnmt3L*-independent retroviral and pericentromeric methylation, which may be mediated by *Hells* activity, is crucial for oocyte meiosis [51]. Further studies on *Hells*-mediated oocyte methylation are required to elucidate the details of this mechanism.

Previous studies on the cytosine methylation of mtDNA have been highly controversial. A recent study by Shock et al. [53] reported cytosine methylation and hydroxymethylation in mammalian mitochondria. Our results indicated that mtDNA is unmethylated in blastocysts and ESCs, but is partially methylated in germ cells. Whether or not 5-hydroxymethylcytosine (5-hmC) exists in mitochondrial or genomic chromosomes of germ cells remains unclear. Meanwhile, rapid hydroxylation of 5-methylcytosine (5-mC) in the paternal pronucleus during zygotic development was also recently reported [54], [55]. Currently, it is difficult to assess hydroxymethylation profiles in oocyte genomes due to the limited DNA recovery. Further investigation of cytosine modification during germ cell and zygote development will be required in the future to better understand this process.

The DNA methylome maps of mouse germ cells, in this study, were derived from SBS data and, therefore, accurately represent methylation levels of individual CpGs on a whole-genome level. The adaptation of the SBS method for small-scale DNA analysis, described in the present report, has the potential to enable further analyses of germline lineages. The current work examined SBS library construction using 3 methods, MethylC-seq, WBA-seq, and PBAT. MethylC-seq basically required only microograms of DNA [22], [23], [56], thus over amplification might cause redundancy in oocyte libraries. The latter methods allow comprehensive methylome analysis in samples with low amounts of starting DNA by avoiding DNA damage due to sodium bisulfite treatment (after adapter ligation, in the case of MethylC-Seq). Recent studies using BS sequencing have shown that methylated cytosine is abundant in the non-CpG regions of human pluripotent stem cells and mouse oocytes [22], [23], [39], [56]; however, the function of non-CpG methylation in mammalian genomes remains unclear. The PBAT results also showed an abundance of non-CpG methylation in oocytes, with results similar to a previous sequencing study on imprinted loci [39]. However, accurate assessment of non-CpG methylation is required using increased sequencing depths because methylation levels of the non-CpG sites were much lower than those of the CpG sites. SBS library construction was conducted by WBA-seq from 2000 fully matured (metaphase II stage) oocytes; sufficient quantities for sequencing were not obtained. During oogenesis, most of the oocyte specific imprinted methylation marks were established during the GV stage. This contrasted to a previous study where a continuous increase in methylation levels was observed [38]. Further improvement of SBS methods, requiring smaller amounts of DNA, is needed to provide complete germ cell methylome maps and to elucidate the exact function of non-CpG methylation in germ cells.

In conclusion, we constructed the first extensive, high-resolution maps of DNA methylomes of mouse oocytes and sperm. These maps described the epigenetic properties of these DNA methylomes. Our data could serve as a platform for future studies to elucidate the role of epigenetic modifications in the development and functioning of germ and stem cells. Such studies are anticipated to improve our understanding of epigenetic reprogramming.

## Materials and Methods

### Preparation of MethylC-seq libraries

Five thousand germinal vesicle (GV)-stage oocytes were collected from the ovarian follicles of adult (7- to 9-week-old) female C57BL/6N mice (Clea Japan, Tokyo, Japan) 44–48 h after they were injected with equine chorionic gonadotropin. Three hundred blastocysts at embryonic day 3.5 were obtained from superovulated adult female C57BL/6N mice by flushing the uterus. Genomic DNA was extracted using the QIAamp DNA Mini Kit (Qiagen, Valencia, CA). Sperm were released from the cauda epididymises of adult male C57BL/6N mice. Sperm DNA was isolated by a standard phenol-chloroform extraction procedure with dithiothreitol (DTT). Genomic DNA from 2 lines of ESCs derived from C57BL/6J mice (Clea Japan) was extracted using the DNeasy Blood & Tissue Kit (Qiagen). DNA samples were sheared into 100-bp fragments in oocytes and 200-bp fragments in other samples using the Covaris S2 focused acoustic system (Covaris, Woburn, MA). Cytosine-methylated adapters (Illumina, San Diego, CA) were ligated to DNA by using the Paired-End DNA Sample Prep Kit or ChIP-Seq DNA Sample Prep Kit (Illumina). DNA fragments were isolated by 2–3% agarose gel electrophoresis and purified using the QIAquick Gel Extraction Kit (Qiagen). Sodium bisulfite conversion was performed using the Epitect Bisulfite Kit (Qiagen).

All bisulfite-converted DNA molecules were polymerase chain reaction (PCR)-amplified as follows: 2.5 U of Hot Start Taq polymerase (TaKaRa, Tokyo, Japan), 5 µL 10× PCR buffer, 25 µM dNTPs, 1 µL of each PCR Primer PE 1.0 and 2.0 (Illumina) (50 µL final). Thermocycling parameters were: initial denaturation at 94°C for 1 min, 15–25 cycles of denaturation at 94°C for 30 s, annealing at 65°C for 30 s, and extension at 72°C for 30 s, followed by a final extension at 72°C for 5 min. PCR reaction products were purified using the QIAquick kit (Qiagen).

### Preparation of whole WBA-seq libraries

Two thousand GV-stage oocytes were collected from 7- to 9-week-old female C57BL/6N mice (Clea Japan) and, 2300 GV-stage oocytes were collected from 7–15-week-old *Dnmt3L^−/−^* female mice (129SvJae×C57BL/6N hybrid genetic background) [6], [57]. Genomic DNA was extracted using the QIAamp DNA Mini Kit (Qiagen), and then bisulfite-treated with Epitect Bisulfite Kit (Qiagen). Subsequently, the bisulfite-converted DNA was amplified using Epitect Whole Bisulfitome Kit (Qiagen). The collected DNA was sheared into 200-bp fragments using Covaris S2. Unmodified Paired-End adapters (Illumina) were ligated to the DNA by using the Paired-End DNA Sample Prep Kit (Illumina). DNA fragments were isolated by 2% agarose gel electrophoresis and purified using the QIAquick Kit (Qiagen). All DNA was PCR amplified and purified in the same manner as the MethylC-seq method, except the number of PCR cycles was reduced to 7.

### Preparation of PBAT libraries

GV-stage oocytes (400) and blastocysts (100) were obtained from 7- to 9-week-old female C57BL/6N mice (Clea Japan), and genomic DNA was extracted using the QIAamp DNA Mini Kit (Qiagen). The isolated oocyte and blastocyst genomic DNA and 100 ng of genomic DNA from sperm, blastocysts, and ESCs containing 1∶200 amount of unmethylated lambda DNA (Invitrogen, Carlsbad, CA) were bisulfite-treated using the MethylCode Bisulfite Conversion Kit (Invitrogen). Details of the PBAT method are unpublished [Miura F & Ito T, personal communication]. Briefly, bisulfite-treated DNA were double-stranded using Klenow Fragments (3′- 5′ exo-) (New England Biolabs, Ipswich, MA) with random primers containing 5′ biotin tags and Illumina PE adaptors. The biotinylated molecules (first strand) were captured using Dynabeads M280 Streptavidin (Invitrogen) and double-stranded using Klenow Fragments (3′-5′ exo-) with random primers containing Illumina PE adaptors (second strand). Finally, template DNA strands were synthesized as complementary DNA with a second strand (unmethylated C is converted to T) using Phusion Hot Start High-Fidelity DNA Polymerase (New England Biolabs) with PCR Primer PE 1.0 (Illumina).

### Preparation of mRNA sequencing libraries

Total RNA from 1000 wild-type GV oocytes, 500 *Dnmt3L^−/−^* GV oocytes, sperm, and ESCs was extracted using the RNeasy Mini Kit (Qiagen) and treated with DNase I (Promega, Madison, WI). RNA-Seq libraries were constructed using the mRNA-Seq Sample Preparation Kit (Illumina).

### Sequencing

The MethylC-seq for blastocysts, WBA-seq, and PBAT libraries were sequenced on a HiSeq 2000 sequencing system (Illumina); the other MethylC-seq and mRNA-seq libraries were sequenced on a Genome Analyzer II (Illumina). Sample preparation, cluster generation, and sequencing were performed using the Paired-End Cluster Generation Kit-HS and the TruSeq SBS Kit-HS for the HiSeq 2000. Similarly, the Paired-End Cluster Generation Kits v2 and v4 and 18- and 36-Cycle Sequencing Kits v3 and v4 were used for the Genome Analyzer II. All kits were from Illumina.

### Gene mapping

All sequenced reads were processed using the standard Illumina base-calling pipeline (v1.4–1.7). Generated sequence tags were mapped onto the mouse genome (mm9, UCSC Genome Browser, July 2007, Build 37.1) by using the Illumina ELAND program.

MethylC-seq tags (36 or 76 nt) were mapped with a custom Perl program, as described previously [17], [22]. Briefly, all cytosines in the tags were replaced by thymines. Next, these tags were aligned to 2 mouse genome reference sequences (mm9), such that the antisense strand had cytosines replaced by thymines and the sense strand had guanines replaced by adenines. Finally, all tags (32–76 nt) that mapped uniquely without any mismatches to both strands were compiled and used for further analyses.

The 76 nt WBA-seq tags were mapped as follows. All tags were converted to 2 types of reads; in 1 read (“For” read), cytosines were replaced by thymines and in the other read (“Rev” read), guanines were replaced by adenines. Both “For” and “Rev” reads were aligned to sense and antisense mm9 strands. A total of 793, 397, 948, 480, and 238 million tags were aligned in wild-type oocytes, *Dnmt3L^−/−^* oocytes, sperm, blastocysts, and ESC genomes, respectively. To avoid bias, tags mapped with multiple hits or matched chromosome M (mitochondria), chromosome Y, or 3 types of repetitive sequences (simple repeat, low complexity repeat, and satellite DNA sequences) were omitted from further analyses.

The 47 nt PBAT tags (trimmed first 4 nt and last 1 nt) were mapped as follows. All guanidines in the tags were replaced by adenines, and these tags were aligned to sense and antisense strands mm9.

For gene-level analysis, the concentrations of the perfectly matching 35 nt (trimmed first nt) mRNA-seq tags from wild-type oocytes, *Dnmt3L^−/−^* oocytes, sperm, and ESCs were calculated for the genomic regions corresponding to those covered by the RefSeq transcript models. The expression level of 20,854 unique genes was ranked by expression levels (calculated as RPKM values) in each library (Table S1). A total of 33, 28, 23, and 25 tags were aligned in 4 mRNA-seq libraries, respectively. mRNA-seq data analysis was performed and visualized using GenomeStudio Data Analysis software (Illumina).

### Methylation analysis

The percentage of individual cytosines methylated at all CpG sites covered by at least 1 read was calculated as 100×(number of aligned cytosines (methylated cytosines))/(total number of aligned cytosines and thymines (originally unmethylated cytosines)). All genomic CpG methylation data are available on our website (http://www.nodai-genome.org/mouse_en.html). The CpG and non-CpG (CpH) methylation levels determined by PBAT results were calculated as the ratio between the total read C and the total read T mapped to genomic cytosines. Bisulfite conversion failure rates were calculated by read C∶T ratios from lambda DNA mapping data. The failure rates were as follows: GV oocyte, 0.009; sperm, 0.008; blastocysts, 0.011; and ESCs, 0.006. Locations of transposable elements in the mouse genome (mm9) were obtained from the UCSC Genome Browser, and the average methylation levels of the whole genome and each transposable element were recalculated from the ratio of the aligned cytosines and thymines in each sequence. Lists of 23,021 CGIs were obtained from a previous report [37]. Around the TSS and TTS (±5 kb), genomic regions were divided into 20-bp bins. For each bin, the average methylation value was calculated for each gene. The expression level of 20,854 genes was divided into 5 percentile groups ranked by RPKM values, and the average methylation level for each group was mapped onto the gene structure model. These computational analyses were performed using a custom Perl program. Supercomputing resources were provided by the Human Genome Center, Institute of Medical Science, University of Tokyo.

### Statistical analysis

Correlations between gene expression ranks and average methylation levels in the promoter (±500 bp from the TTS) or gene-body regions (gene-body 1: +2 to +5 kb from the TSS; gene-body 2: 0 to −5 kb from the TTS) were calculated using Spearman's rank correlation coefficient (ρ). An R-squared value (R^2^) was calculated to evaluate the correlation of RPKM values between wild-type and *Dnmt3L^−/−^* oocytes. Statistical analysis was performed using the R statistical package.

### Bisulfite sequencing

To analyze the methylation of the three transposable elements (L1 LINE, B1/Alu SINE, and IAP LTR), 20 wild-type GV oocytes were obtained from adult female C57BL/6N mice. Bisulfite sequencing conditions and primer sets for the three transposable elements were described, previously [52]. To analyze the methylation of the *Gpr1* locus, 10 blastocysts were obtained from BJF1 (C57BL/6N×JF1) and *Dnmt3L^mat−/−^* (*Dnmt3L^−/−^*×JF1) mice [6], [57]. Genomic DNA from blastocysts was isolated using the QIAamp DNA Mini Kit (Qiagen) and treated with sodium bisulfite with the Epitect Bisulfite Kit (Qiagen). The *Gpr1* gDMR sequence was amplified with 2 rounds of nested PCR. The first-round PCR reaction contained 1 U of Hot Start Taq polymerase (TaKaRa), 1× PCR buffer, 200 µM dNTPs, 1 µM forward primer, and 1 µM reverse primer (20 µL final). Thermocycling parameters were as follows: initial denaturation at 94°C for 1 min, 35 cycles of denaturation at 94°C for 30 s, annealing at 50°C for 30 s, and extension at 72°C for 30 s, followed by a final extension at 72°C for 5 min. Subsequently, 2 µL of the product was used as the input for the second-round PCR, which was performed in the same manner. Primer sets for the nested PCR were as follows: Gpr1-BSF1 (5′-GATTAGATTAGGTTAGTTTGGAA-3′) and Gpr1-BSR1 (5′-ACTAAAACACTAATCACCAAATA-3′) for the first round; Gpr1-BSF2 (5′-AGATTAGGTTAGTTTGGAATT-3′) and Gpr1-BSR2 (5′-AACACTAATCACCAAATAATTC-3′) for the second round. The second-round PCR product was subcloned and sequenced, as described previously [50]. The percentage methylation was calculated as 100×(number of methylated CpG dinucleotides)/(total number of CpGs). At least 10 clones from each parental allele were sequenced. Sequence data were analyzed using the QUMA quantification tool for methylation analysis [58].

### Accession number

The MethylC-seq, WBA-seq, PBAT, and mRNA-seq data in this study have been deposited in the DNA Data Bank of Japan (DDBJ) under accession number DRA000484.

## Supporting Information

Figure S1 Schematic of the SBS library construction procedure. MethylC-Seq libraries were generated by ligation of methylated sequencing adapters to fragmented genomic DNA followed by gel purification, sodium bisulfite conversion, and PCR amplification (*left*). WBA-seq libraries were generated by ligation of unmodified sequencing adapters to bisulfite-modified (amplified using EpiTect Whole Bisulfitome Kits) and fragmented genomic DNA followed by gel purification and PCR amplification (*middle*). PBAT libraries were generated by double-stranded DNA synthesis from bisulfite-treated (single-stranded) DNA with random primers containing sequencing adapters (*right*).
(TIF)

Figure S2 The percent of the oocyte and sperm genomes covered by differing minimum numbers of MethylC-seq and WBA-seq reads.
(TIF)

Figure S3 Sequencing bias towards mitochondrial and repetitive DNA sequences. (A) Average read depths for autosomal chromosomes and chromosome M (mitochondria) of mouse oocyte and sperm genomes. Occupancy of transposable elements in reads from SBS libraries before (B) and after (C) filtering the biased reads. (D) Genomic CpG coverage of SBS reads for each chromosome of mouse oocyte (orange: MethylC-seq, red: combined between MethylC-seq and WBA-seq) and sperm genomes (blue).
(TIF)

Figure S4 Average CpG methylation levels in genomic chromosomal DNA and mitochondrial DNA.
(TIF)

Figure S5 High-resolution DNA methylome map on mouse X inactivation center region in chromosome X (100,200,000–101,200,000). GenomeStudio view of Refseq's positions, repetitive element, CpG methylation map, CpG densities, CGI positions, and CGI methylation map were shown. Red, purple, blue, green, and khaki dots and boxes represent the methylation levels at individual CpGs and CGIs in wild-type oocyte, *Dnmt3L* ^−/−^ oocyte, sperm, blastocyst, and ESC genomes, respectively, as shown in Figure 1.
(TIF)

Figure S6 DNA methylome maps of each chromosome of mouse germ cells. The methylation levels of each chromosome in wild-type oocytes, *Dnmt3L^−/−^* oocytes, and sperm in 10 kb windows (excluding mitochondrial chromosome, chromosome Y, and unplaced contigs). Red, purple, and blue lines represent the methylation levels in wild-type oocytes, *Dnmt3L^−/−^* oocytes, and sperm, respectively. Red and blue boxes represent oocyte-methylated and sperm-methylated gDMRs, and red and blue pins indicate maternal and maternal ICRs, respectively.
(TIF)

Figure S7 Methylation profiling of transposable elements in mouse germ cells. (A) CpG methylation levels are plotted as a function of CpG densities for L1 LINE, B1/Alu SINE, and LTR/ERVK retrotransposons (approximately 10% of the latter are intracisternal A particle (IAP) LTRs). Data for high CpG densities including less than 100 genomic CpGs were not plotted. (B) Bisulfite sequencing of L1 LINE, B1/Alu SINE, and IAP LTR retrotransposons. Methylated and unmethylated CpGs are indicated by open and closed circles, respectively.
(TIF)

Figure S8 Transcriptome and DNA methylome profiling at *H19-Igf2*, GenomeStudio view of mRNA-seq data (*top*) and CpG methylation map (*bottom*) of the genomic region spanning each locus. The blue shaded areas show the extent of the paternally-methylated gDMR.
(TIF)

Figure S9 Transcriptome and DNA methylome profiling at *Dnmt1*. The red shaded areas show the extent of the maternally-methylated gDMR.
(TIF)

Figure S10 Transcriptome and DNA methylome profiling at *Gpr1-Zdbf2*. The blue and red shaded areas show the extent of the paternally- and maternally-methylated gDMRs, respectively.
(TIF)

Figure S11 Quantification of the ratio of methylated (total number of read C) versus unmethylated cytosines (total number of read T) by PBAT results. Bar charts represent cytosine methylation ratio (A) at CpG (*left*), CpHpG (*middle*), and CpHpH (*right*) contexts and bisulfite-conversion failure rate (B) calculated by C∶T ratio from lambda DNA mapping data. Total number of mapped reads is shown on these charts (*Top*).
(TIF)

Figure S12 Relationship between gene expression and intragenic methylation in ESCs. (A) The expression level of genes in ESCs was divided into 5 percentile groups. The distribution of methylation is shown ±5 kb from the transcription termination site (TTS; *left*) and transcription start site (TSS; *middle*). The graphs on the right show the average methylation levels in the promoter and gene-body regions. Spearman's rank correlation coefficient (ρ) was used to test the statistical significance of the correlation between gene expression and DNA methylation levels (*: p<1×10^−9^).
(TIF)

Figure S13 Expression profiles of DNA methyltransferase gene families. Red, purple, blue, and khaki bars represent RPKM values of individual genes in wild-type oocytes, *Dnmt3L^−/−^* oocytes, sperm, and ESCs.
(TIF)

Figure S14 Expression profiles of pluripotency-associated genes among wild-type oocytes, *Dnmt3L^−/−^* oocytes, sperm, and ESCs.
(TIF)

Figure S15 Histograms of the methylation levels of the demethylation-resistant (*left*) and demethylation-sensitive gDMRs (*right*) in ESCs. The number of oocyte-specific and sperm-specific methylated gDMRs is shown in red and blue, respectively.
(TIF)

Table S1 Gene transcript profiling for germ cells, blastocysts, and embryonic stem cells by mRNA-seq.
(XLSX)

Table S2 DNA methylation profiles of 23,021 CGIs.
(XLSX)

Table S3 Average DNA methylation profiles of 646 gDMRs determined by SBS and RRBS methods.
(XLSX)
