# Supplementary figures and images for: Contribution of Intragenic DNA Methylation in Mouse Gametic DNA Methylomes to Establish Oocyte-Specific Heritable Marks
Source: PLoS Genet. 2012 Jan 5;8(1):e1002440. doi: 10.1371/journal.pgen.1002440 (PMC3252278; doi:10.1371/journal.pgen.1002440)

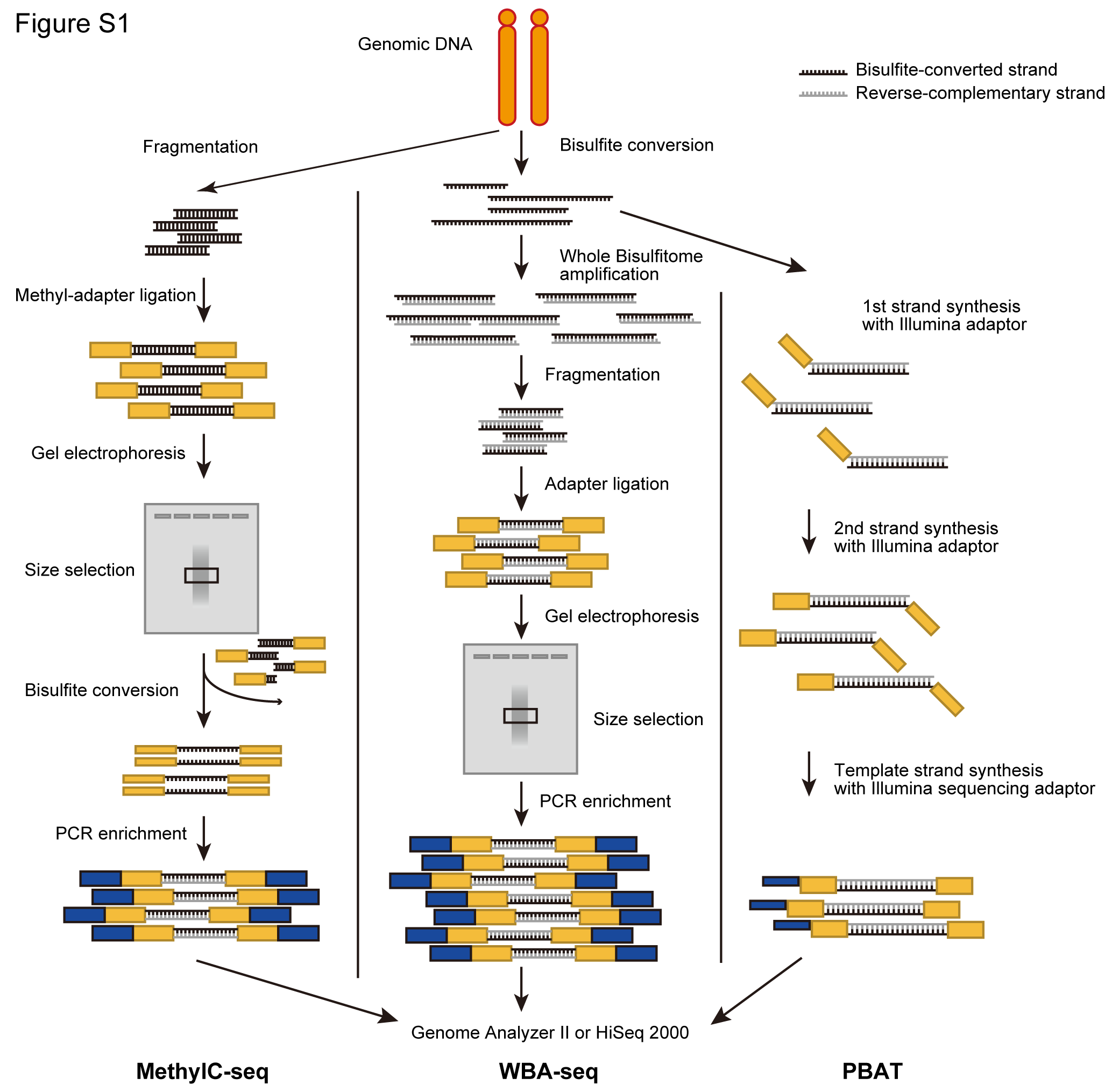

Supplement: Figure S1 — Schematic of the SBS library construction procedure. MethylC-Seq libraries were generated by ligation of methylated sequencing adapters to fragmented genomic DNA followed by gel purification, sodium bisulfite conversion, and PCR amplification (left). WBA-seq libraries were generated by ligation of unmodified sequencing adapters to bisulfite-modified (amplified using EpiTect Whole Bisulfitome Kits) and fragmented genomic DNA followed by gel purification and PCR amplification (middle). PBAT libraries were generated by double-stranded DNA synthesis from bisulfite-treated (single-stranded) DNA with random primers containing sequencing adapters (right). (TIF) [file pgen.1002440.s001.tif]

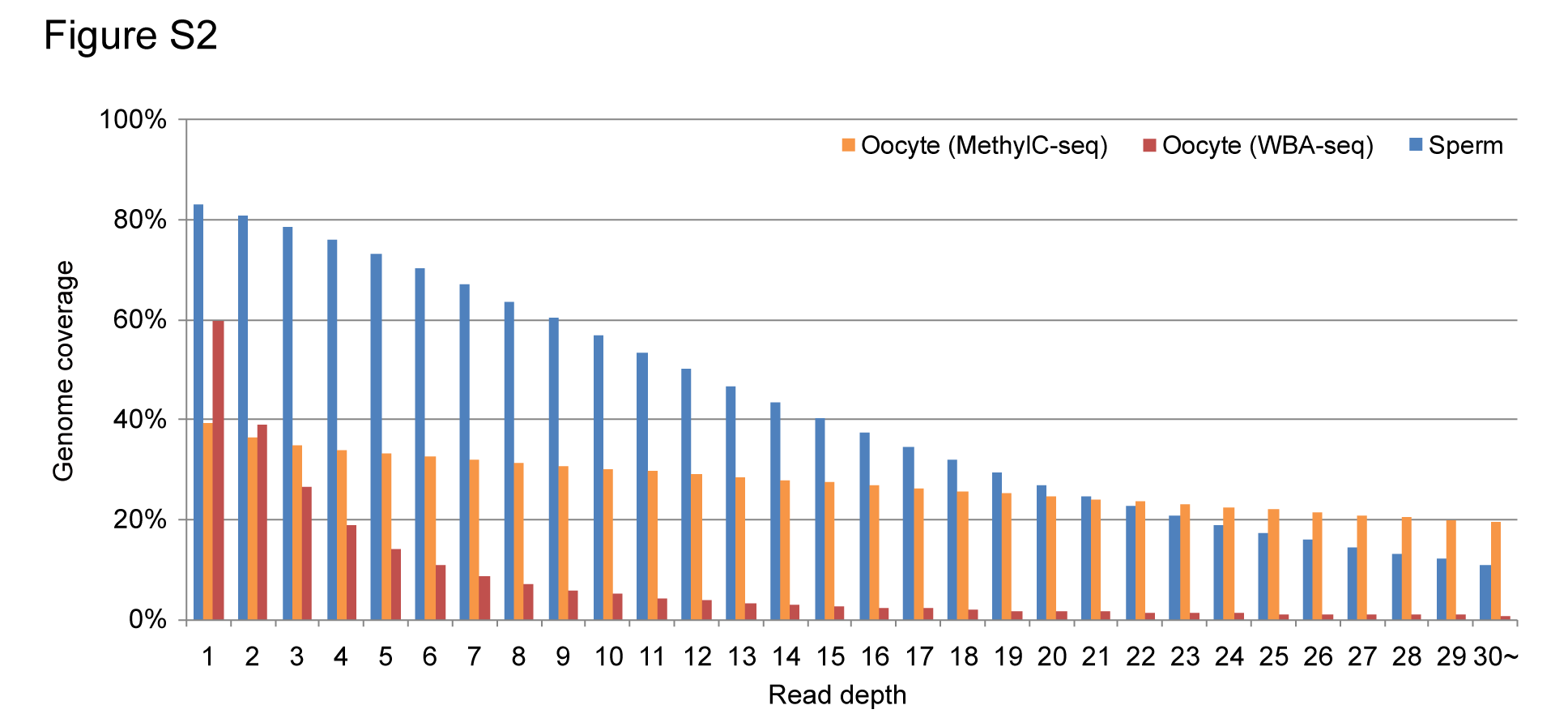

Supplement: Figure S2 — The percent of the oocyte and sperm genomes covered by differing minimum numbers of MethylC-seq and WBA-seq reads. (TIF) [file pgen.1002440.s002.tif]

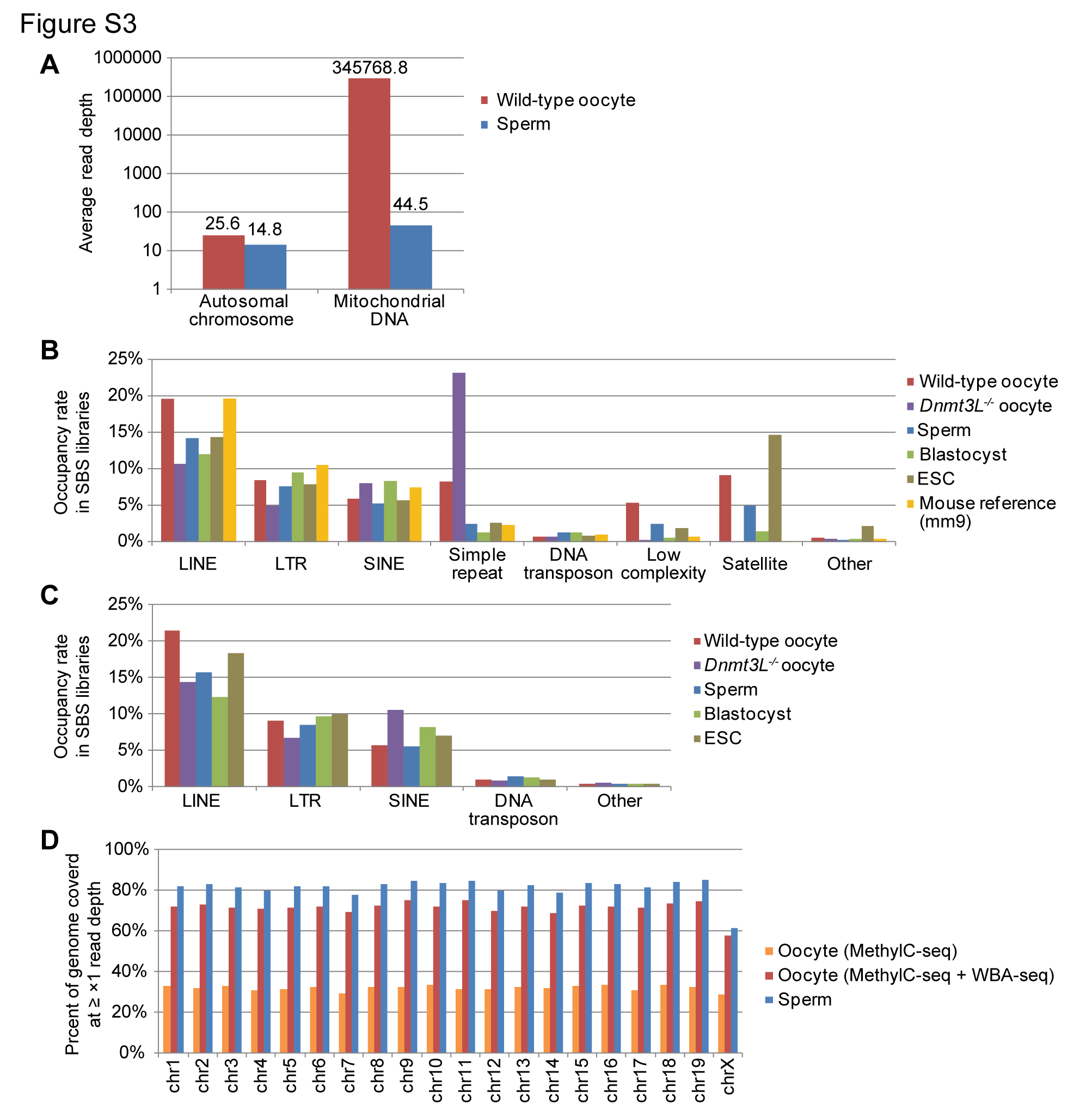

Supplement: Figure S3 — Sequencing bias towards mitochondrial and repetitive DNA sequences. (A) Average read depths for autosomal chromosomes and chromosome M (mitochondria) of mouse oocyte and sperm genomes. Occupancy of transposable elements in reads from SBS libraries before (B) and after (C) filtering the biased reads. (D) Genomic CpG coverage of SBS reads for each chromosome of mouse oocyte (orange: MethylC-seq, red: combined between MethylC-seq and WBA-seq) and sperm genomes (blue). (TIF) [file pgen.1002440.s003.tif]

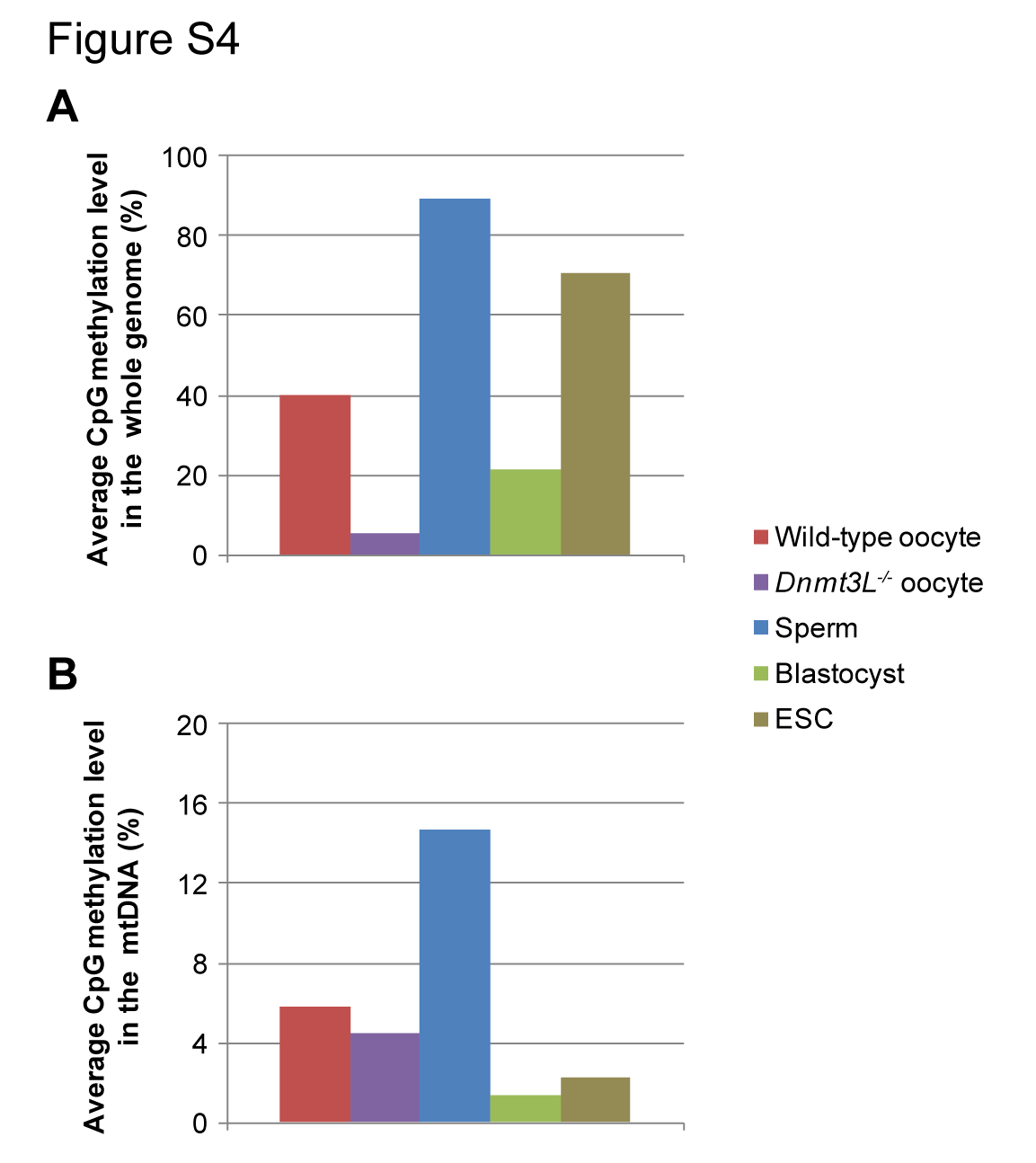

Supplement: Figure S4 — Average CpG methylation levels in genomic chromosomal DNA and mitochondrial DNA. (TIF) [file pgen.1002440.s004.tif]

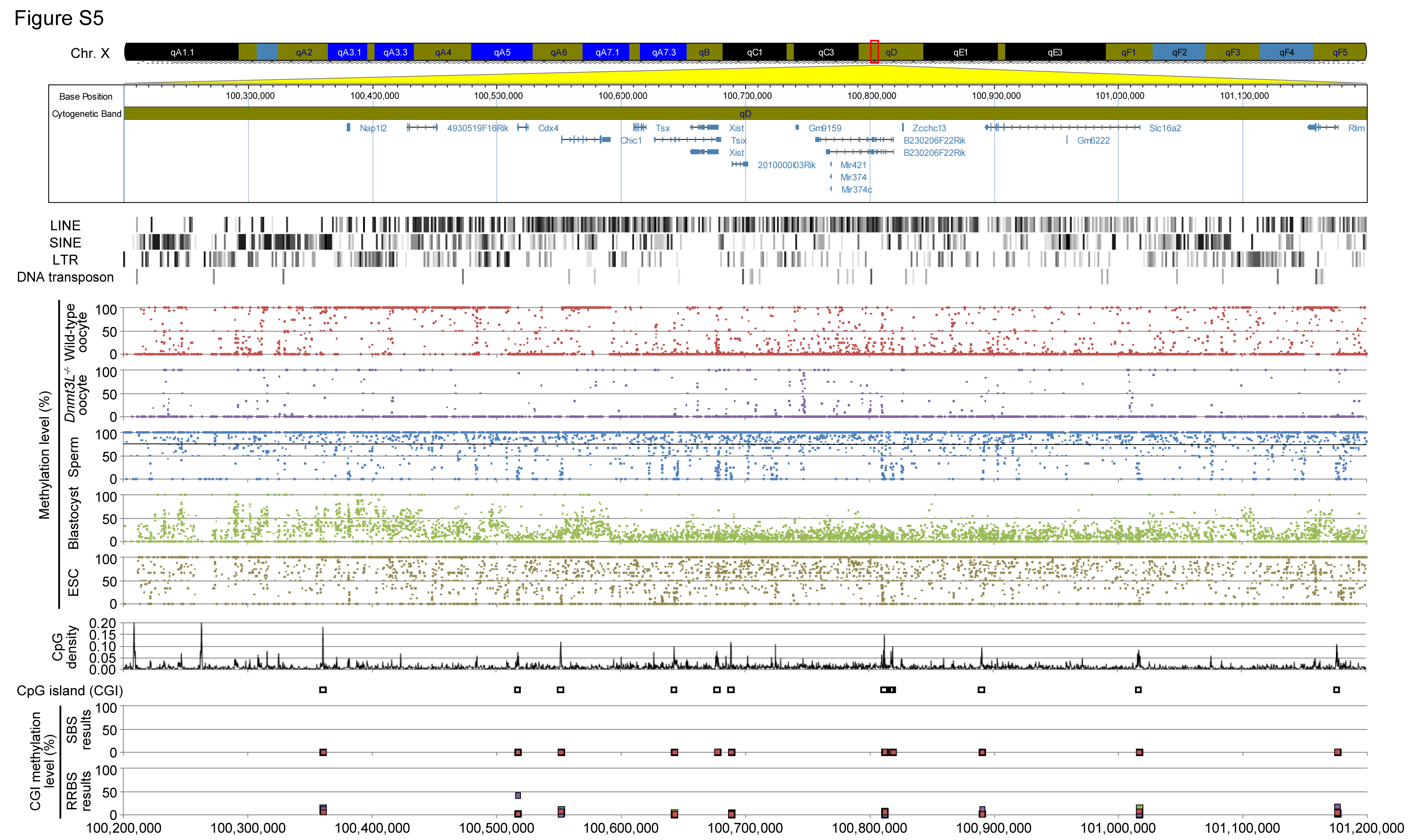

Supplement: Figure S5 — High-resolution DNA methylome map on mouse X inactivation center region in chromosome X (100,200,000–101,200,000). GenomeStudio view of Refseq's positions, repetitive element, CpG methylation map, CpG densities, CGI positions, and CGI methylation map were shown. Red, purple, blue, green, and khaki dots and boxes represent the methylation levels at individual CpGs and CGIs in wild-type oocyte, Dnmt3L −/− oocyte, sperm, blastocyst, and ESC genomes, respectively, as shown in Figure 1. (TIF) [file pgen.1002440.s005.tif]

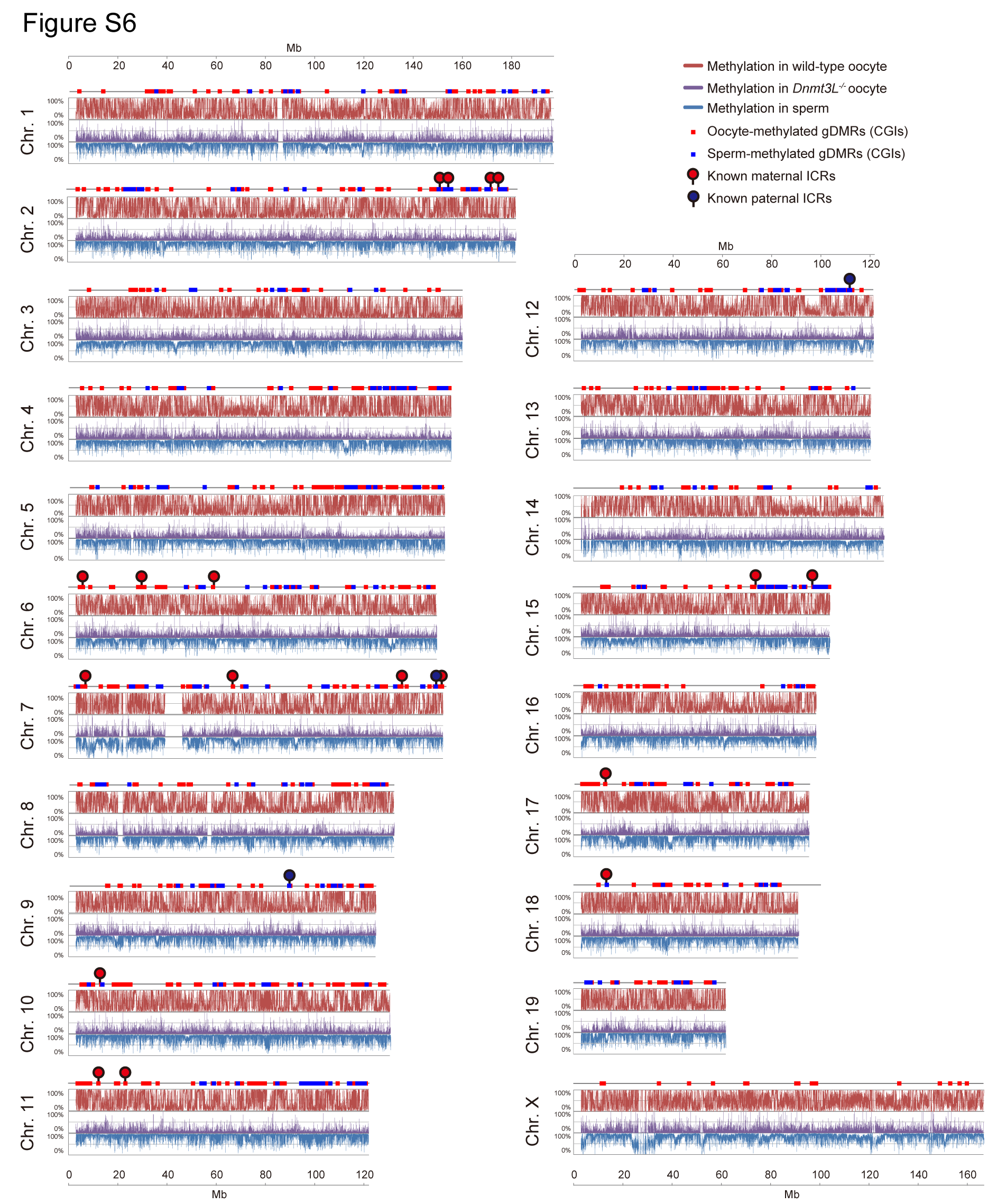

Supplement: Figure S6 — DNA methylome maps of each chromosome of mouse germ cells. The methylation levels of each chromosome in wild-type oocytes, Dnmt3L−/− oocytes, and sperm in 10 kb windows (excluding mitochondrial chromosome, chromosome Y, and unplaced contigs). Red, purple, and blue lines represent the methylation levels in wild-type oocytes, Dnmt3L−/− oocytes, and sperm, respectively. Red and blue boxes represent oocyte-methylated and sperm-methylated gDMRs, and red and blue pins indicate maternal and maternal ICRs, respectively. (TIF) [file pgen.1002440.s006.tif]

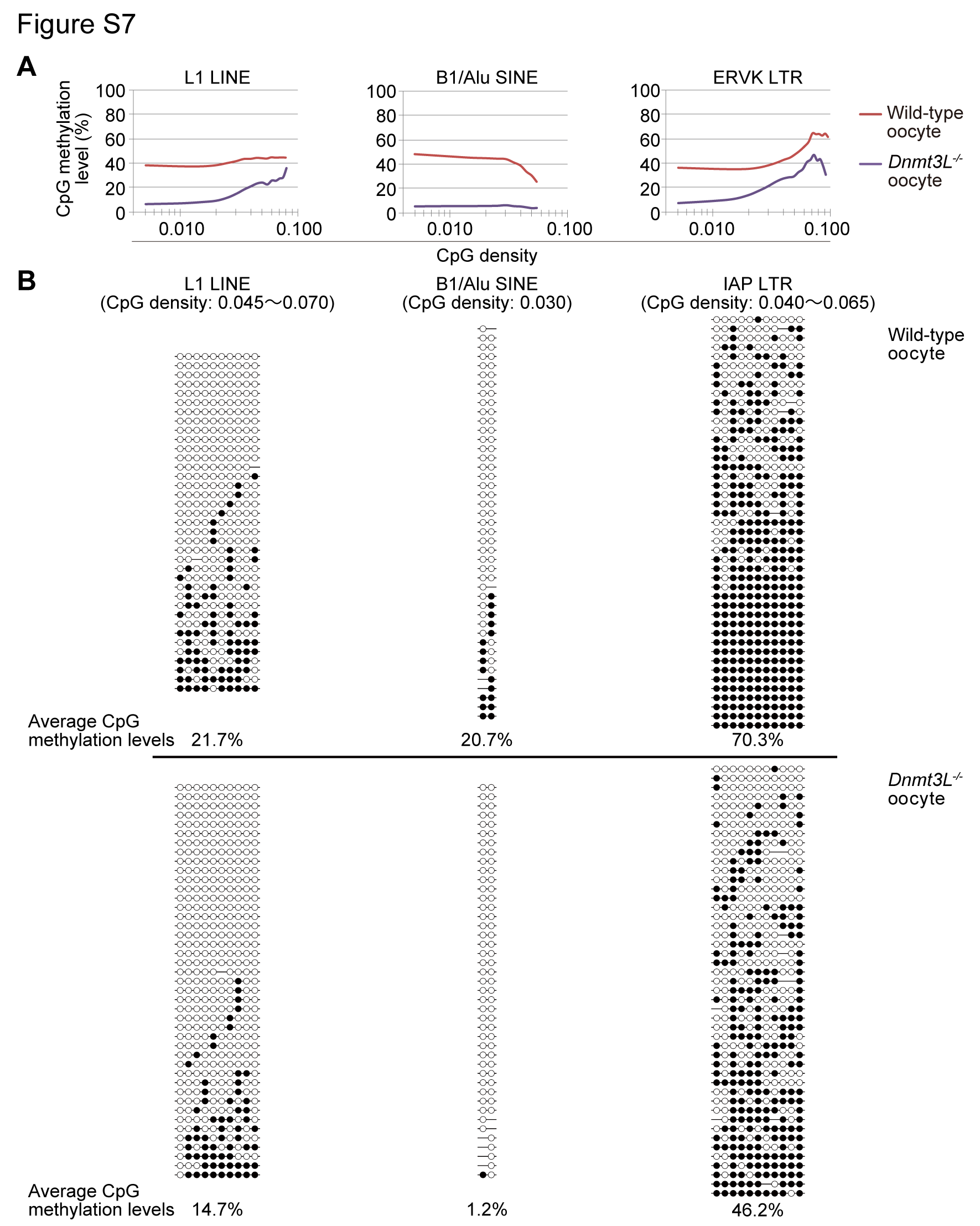

Supplement: Figure S7 — Methylation profiling of transposable elements in mouse germ cells. (A) CpG methylation levels are plotted as a function of CpG densities for L1 LINE, B1/Alu SINE, and LTR/ERVK retrotransposons (approximately 10% of the latter are intracisternal A particle (IAP) LTRs). Data for high CpG densities including less than 100 genomic CpGs were not plotted. (B) Bisulfite sequencing of L1 LINE, B1/Alu SINE, and IAP LTR retrotransposons. Methylated and unmethylated CpGs are indicated by open and closed circles, respectively. (TIF) [file pgen.1002440.s007.tif]

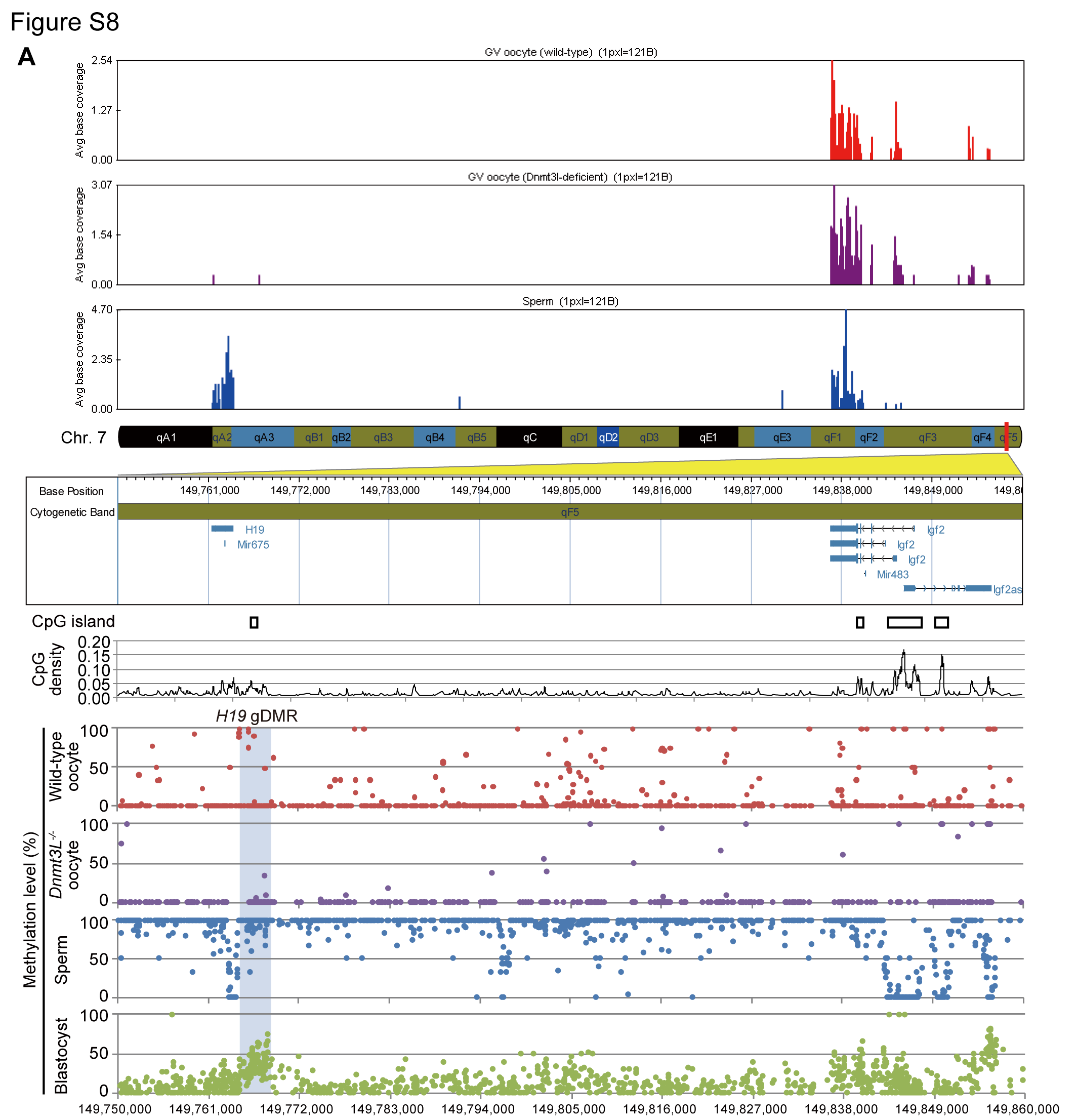

Supplement: Figure S8 — Transcriptome and DNA methylome profiling at H19-Igf2, GenomeStudio view of mRNA-seq data (top) and CpG methylation map (bottom) of the genomic region spanning each locus. The blue shaded areas show the extent of the paternally-methylated gDMR. (TIF) [file pgen.1002440.s008.tif]

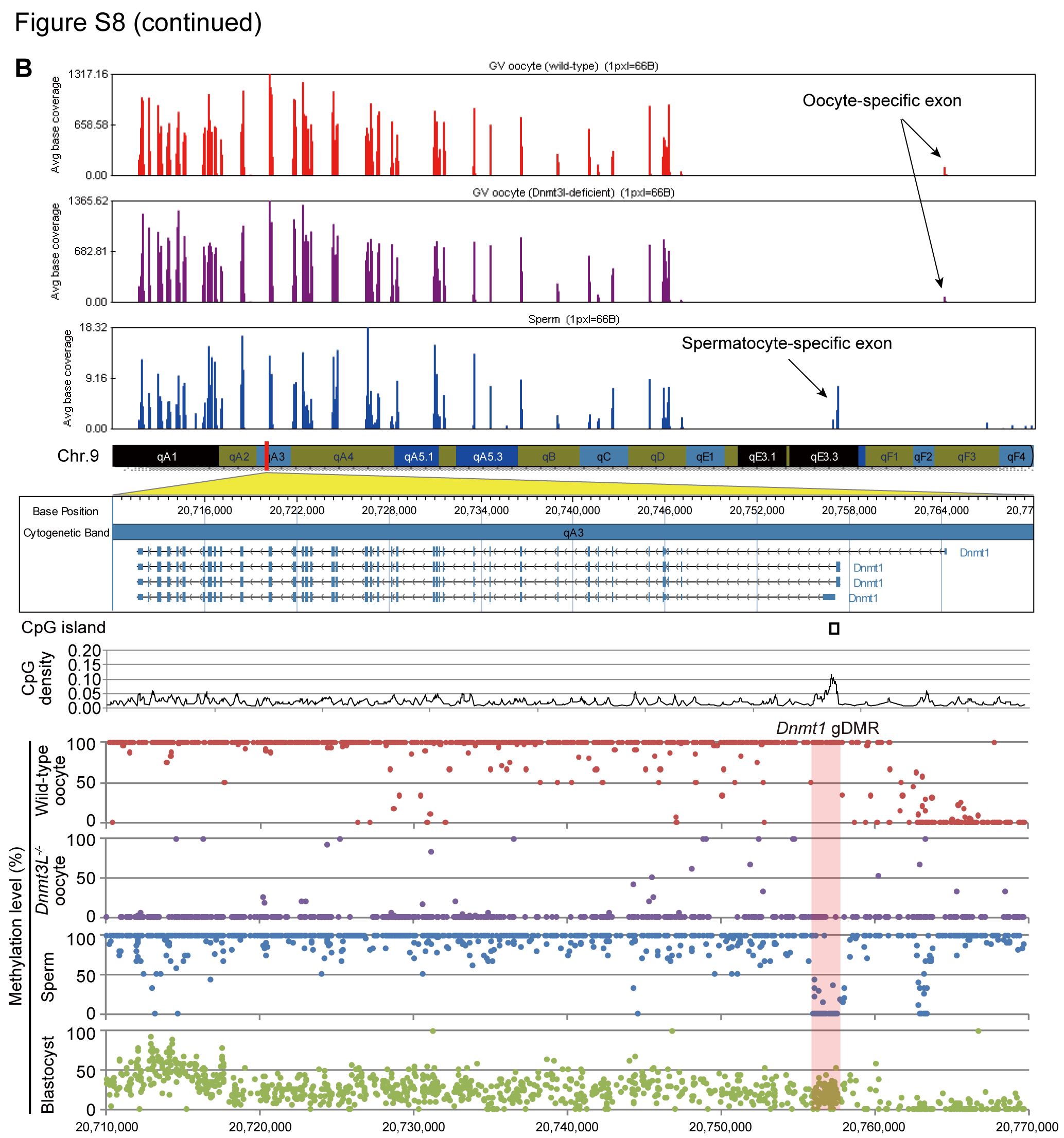

Supplement: Figure S9 — Transcriptome and DNA methylome profiling at Dnmt1. The red shaded areas show the extent of the maternally-methylated gDMR. (TIF) [file pgen.1002440.s009.tif]

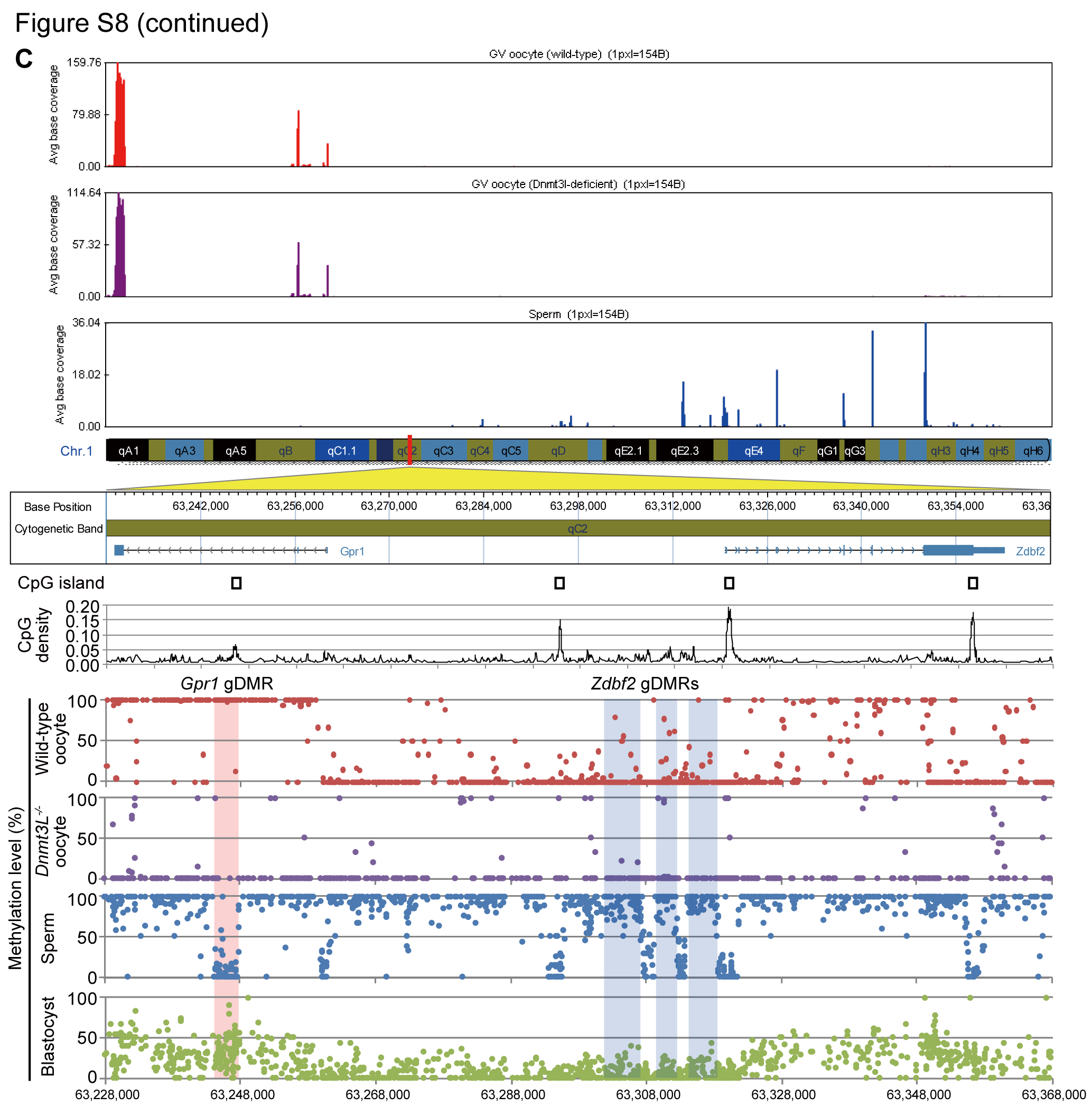

Supplement: Figure S10 — Transcriptome and DNA methylome profiling at Gpr1-Zdbf2. The blue and red shaded areas show the extent of the paternally- and maternally-methylated gDMRs, respectively. (TIF) [file pgen.1002440.s010.tif]

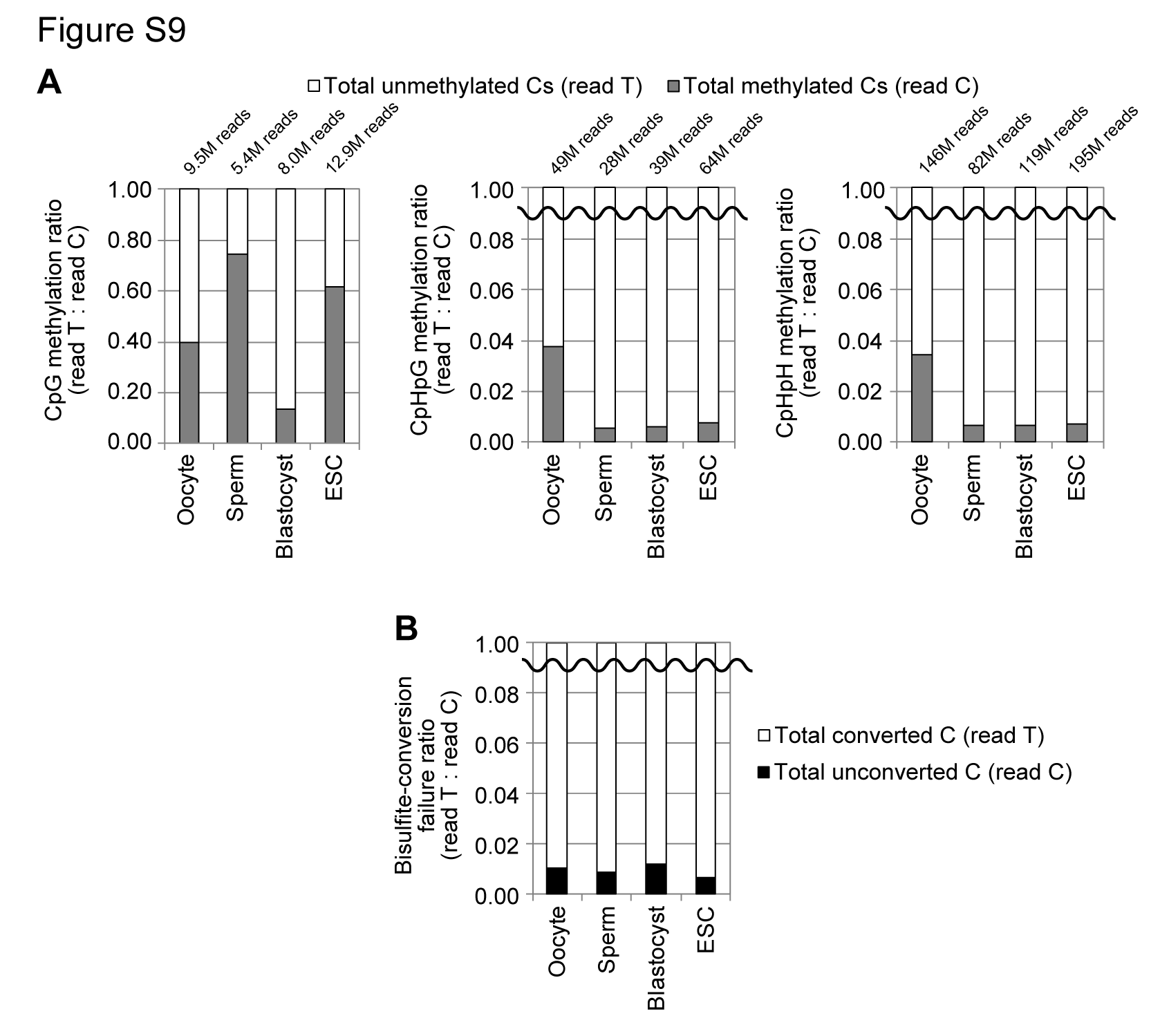

Supplement: Figure S11 — Quantification of the ratio of methylated (total number of read C) versus unmethylated cytosines (total number of read T) by PBAT results. Bar charts represent cytosine methylation ratio (A) at CpG (left), CpHpG (middle), and CpHpH (right) contexts and bisulfite-conversion failure rate (B) calculated by C∶T ratio from lambda DNA mapping data. Total number of mapped reads is shown on these charts (Top). (TIF) [file pgen.1002440.s011.tif]

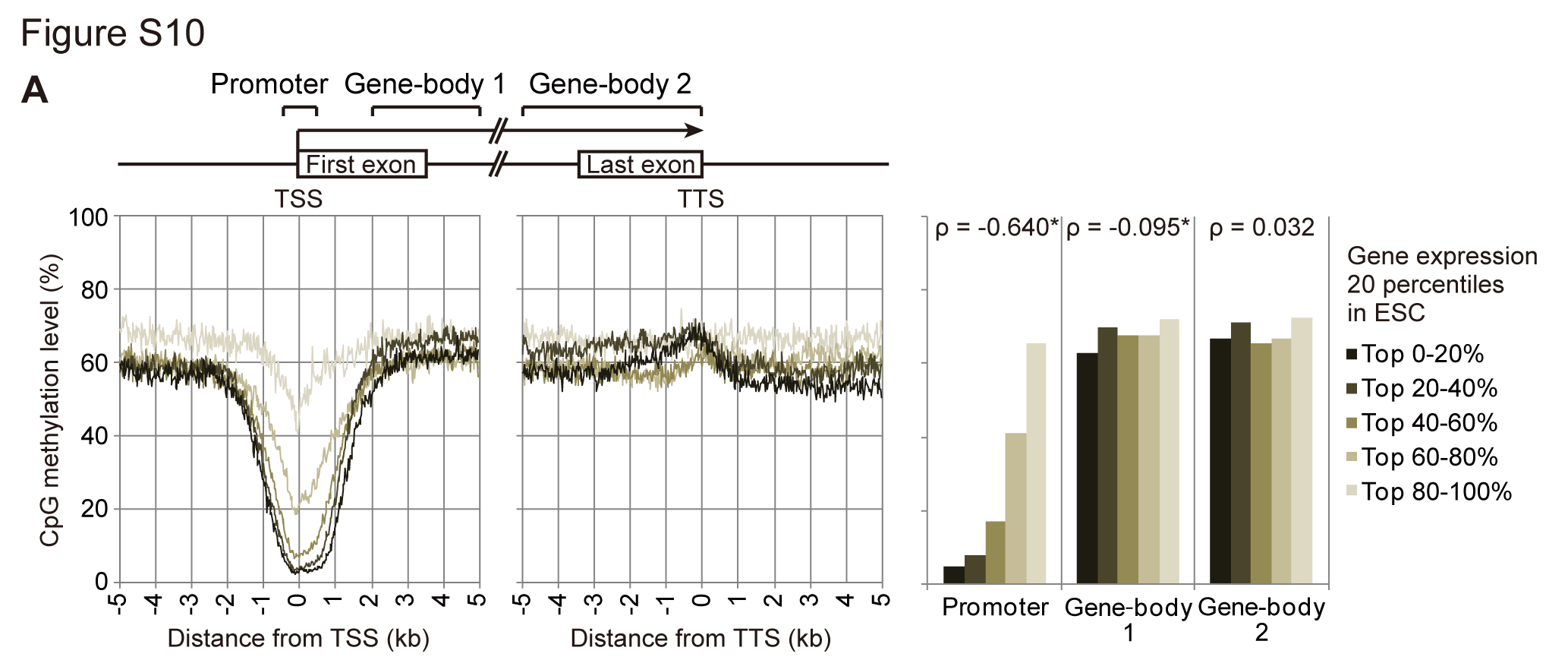

Supplement: Figure S12 — Relationship between gene expression and intragenic methylation in ESCs. (A) The expression level of genes in ESCs was divided into 5 percentile groups. The distribution of methylation is shown ±5 kb from the transcription termination site (TTS; left) and transcription start site (TSS; middle). The graphs on the right show the average methylation levels in the promoter and gene-body regions. Spearman's rank correlation coefficient (ρ) was used to test the statistical significance of the correlation between gene expression and DNA methylation levels (*: p<1×10^−9^). (TIF) [file pgen.1002440.s012.tif]

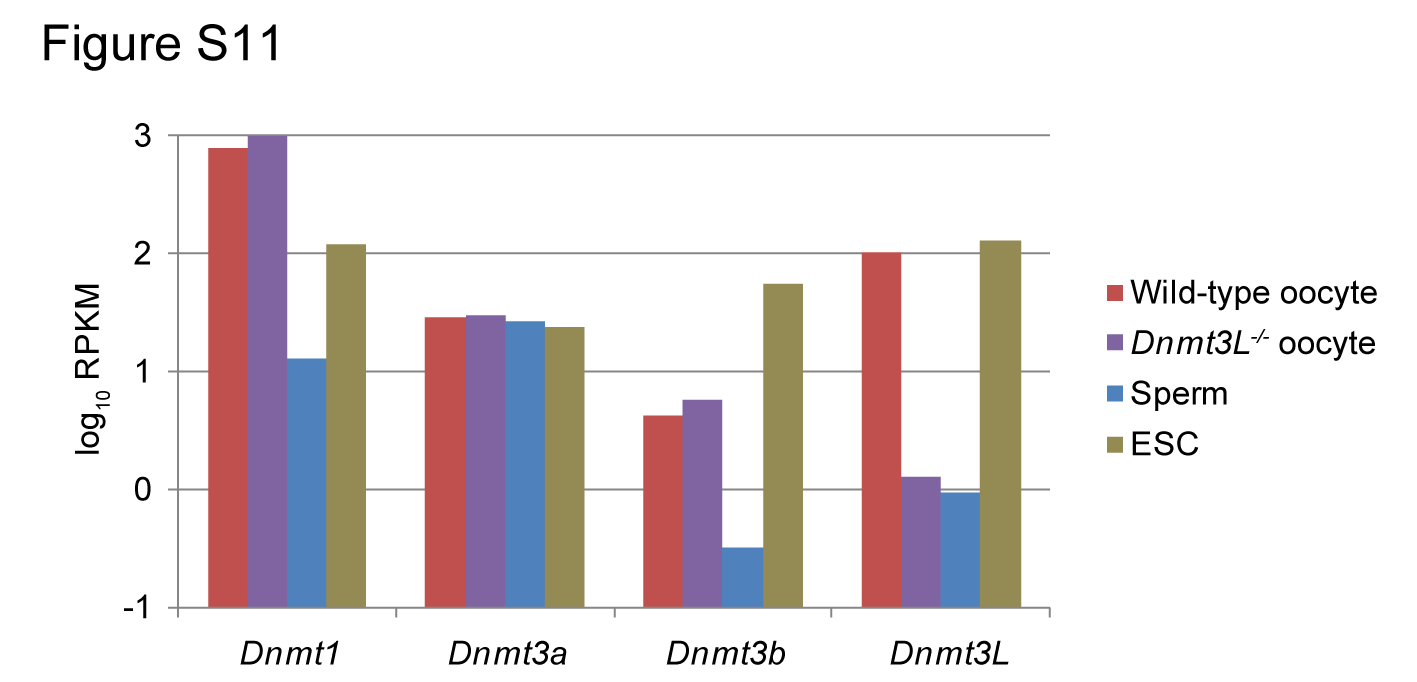

Supplement: Figure S13 — Expression profiles of DNA methyltransferase gene families. Red, purple, blue, and khaki bars represent RPKM values of individual genes in wild-type oocytes, Dnmt3L−/− oocytes, sperm, and ESCs. (TIF) [file pgen.1002440.s013.tif]

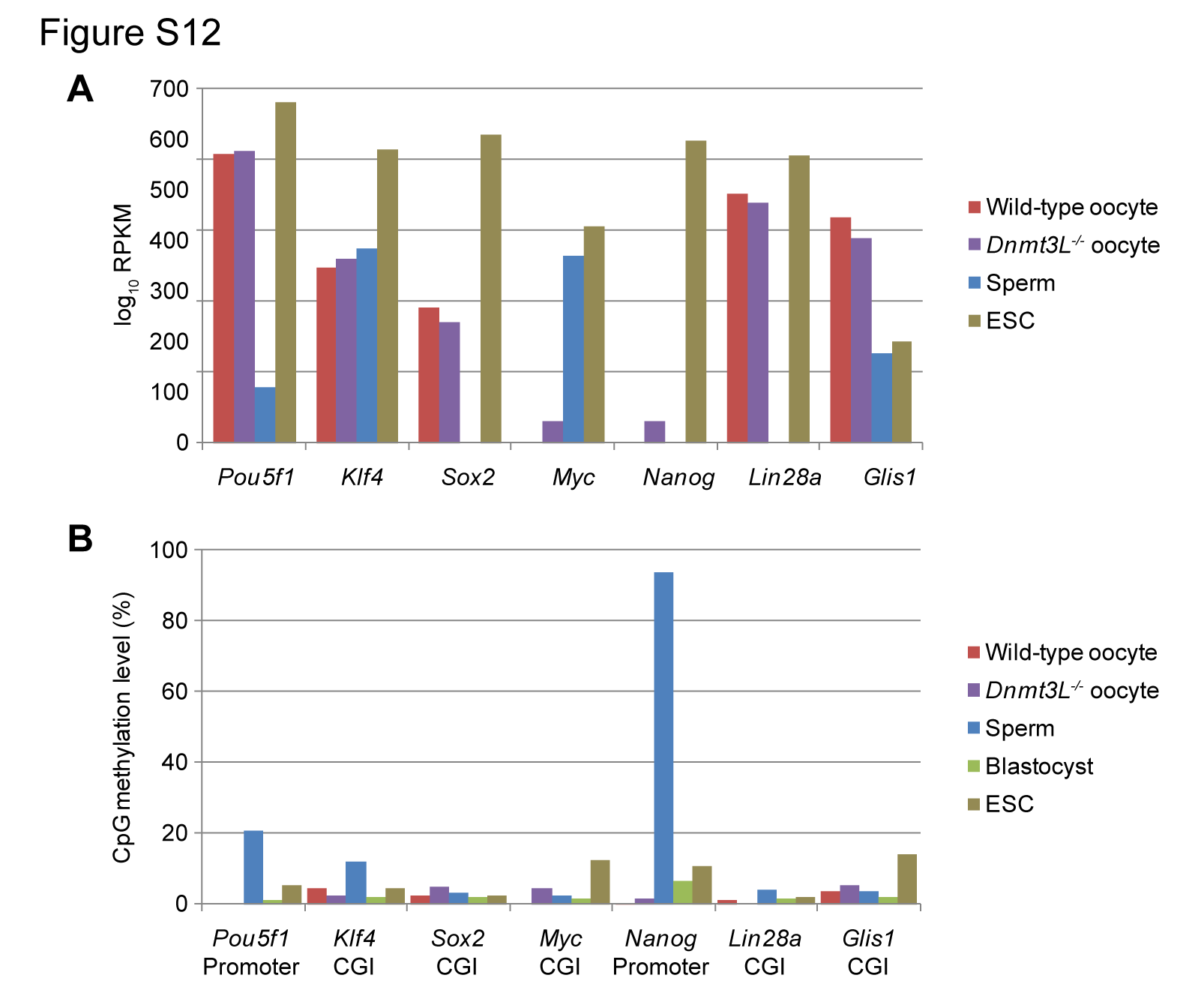

Supplement: Figure S14 — Expression profiles of pluripotency-associated genes among wild-type oocytes, Dnmt3L−/− oocytes, sperm, and ESCs. (TIF) [file pgen.1002440.s014.tif]

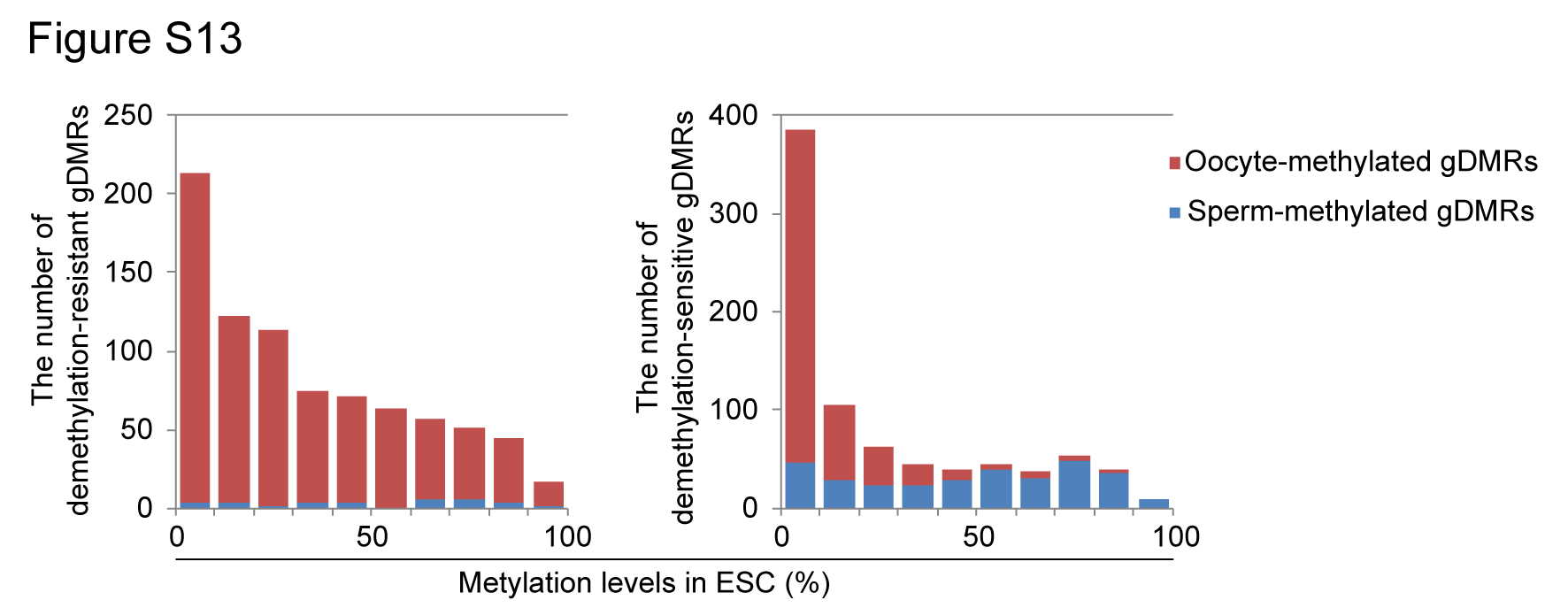

Supplement: Figure S15 — Histograms of the methylation levels of the demethylation-resistant (left) and demethylation-sensitive gDMRs (right) in ESCs. The number of oocyte-specific and sperm-specific methylated gDMRs is shown in red and blue, respectively. (TIF) [file pgen.1002440.s015.tif]
